# Cathepsin L-containing exosomes from α-synuclein-activated microglia induce neurotoxicity through the P2X7 receptor

**DOI:** 10.1038/s41531-022-00394-9

**Published:** 2022-10-06

**Authors:** Tianfang Jiang, Chuanying Xu, Shane Gao, Jia Zhang, Jia Zheng, Xiaolin Wu, Qiuyun Lu, Limei Cao, Danjing Yang, Jun Xu, Xu Chen

**Affiliations:** 1 Department of Neurology, Shanghai Eighth People’s Hospital, 8 Caobao Road, Shanghai, China; 2 Department of Neurology, The Affiliated Hospital of Xuzhou Medical University, 99 West Huaihai Road, Xuzhou, Jiangsu Province China; 3 Department of Neurosurgery, Shanghai East Hospital Affiliated to Tongji University, 150 Jimo Road, Shanghai, China; 4 School of Medicine, Tongji University, 1239 Siping Road, Shanghai, China

**Keywords:** Cellular neuroscience, Parkinson's disease

## Abstract

Uncontrolled microglial activation is pivotal to the pathogenesis of Parkinson’s disease (PD), which can secrete Cathepsin L (CTSL) to affect the survival of neurons in the PD patients; however, the precise mechanism has yet to be determined. We demonstrated for the first time that CTSL was mostly released by exosomes derived from α-Syn-activated microglia, resulting in neuronal damage and death. The elevation of CTSL activity was blocked by GW4869, suggesting a critical role for exosomes in mediating CTSL release. Furthermore, the P2X7R/PI3K/AKT signalling pathway was identified as the underlying molecular mechanism since specific antagonists of this signalling pathway, P2X7R knockdown and exosome release inhibitors significantly reduced the injury to cultured mouse cortical neurons. Our study suggests that increased extracellular release of CTSL from α-Syn-activated microglia through exosomes amplifies and aggravates of the neurotoxic effect of microglia, implying that CTSL may be involved in a fresh mechanism of PD pathogenesis, and serve as a potential biomarker and a target for PD drug development.

## Introduction

It has become evident that among the known pathological processes associated with Parkinson’s disease (PD), excessive microglial activation is undoubtedly a major culprit and that aggregation of a large number of activated microglia in the substantia nigra pars compacta (SNpc), which can be observed in the early phase of PD, is one of the main pathological manifestations of the disease^1,2^ and is the primary source of inflammatory factors and reactive oxygen species^3^. Nevertheless, the key molecular mechanisms that lead to microglial neurotoxicity in PD are poorly defined. The insoluble oligomer form of α-synuclein (α-Syn), a key protein that is strongly implicated in the pathogenesis of PD, is capable of activating microglia. This process is enhanced by missense mutations of α-Syn, especially the A53T mutation^4^. Therefore, exploring the specific mechanism by which α-Syn-activated microglia exert and amplify their toxic effects is very important for identifying new strategies to prevent and treat PD.

Cathepsin L (CTSL), a member of the cysteine protease family, is a partially secreted extracellular protein^5^. Autopsy results have confirmed that the expression level of CTSL in the substantia nigra of PD patients is markedly increased^6^, and peripheral blood test results have also indicated that the mRNA level of CTSL in PD patients is significantly higher than that in normal people^7^. A large increase in CTSL levels is observed after stimulation of microglia in PD models^7–9^ and followed by an inflammatory outbreak, which may further promote apoptosis of neurons^10^, affect axonal growth^11^, and lead to neuronal death. These effects can be blocked by suppressing CTSL, such as by using CTSL inhibitors. Therefore, it is imperative to understand the relationship between microglia and CTSL-mediated neurotoxicity. Our previous studies indicated a potential role for the purinergic P2X7 receptor (P2X7R), which mediates the substantial activation of microglia by α-Syn oligomers^4^. Some studies have further suggested that P2X7R might affect the function of lysosomes and increase the release of cathepsin^12–14^ through promotion of the phosphoinositide 3-kinase (PI3K)/protein kinase B (AKT) signalling pathway^15^. Here, we aimed to explore the release pattern of CTSL from α-Syn-activated microglia and determine whether P2X7R is involved in this process.

Exosomes are unconventional small cellular secretory vesicles with a diameter of 30-100 nm that originate from the endosomal system and are released into the extracellular milieu; exosomes usually act as mediators to promote cell-to-cell communication^16–18^ and can be released in large quantities from various cell types in the central nervous system (CNS), including microglia^19^. Intriguingly, microglia-derived exosomes are capable of transferring nucleic acids, proteins and lipids, etc., regulating the extracellular environment and influencing the normal function of neurons in a large surrounding area^20,21^. It has been confirmed that exosomes released by microglia can be internalised by neighbouring neurons through endocytosis. After exosomes are stereotaxically injected into the mouse brain, pathogenic proteins carried by these exosomes can be detected in neurons of the recipient animal^22,23^. However, the role of exosomes in the pathogenesis of neurodegenerative disorders, especially PD, remains to be elucidated. P2X7R plays some role in regulating the release of exosomes^24^. Dubyak and Barberà et al. observed that activation of P2X7R by adenosine triphosphate (ATP) can lead to the secretion of various cytokines and proteins from cells in the form of exosomes, which amplifies neurotoxicity^25–27^. Proteins carried by exosomes can also serve as useful biomarkers for PD, implying a potential association between exosomes and CTSL.

In this study, we studied the putative roles of CTSL in the pathogenesis and development of PD. We elucidated that CTSL released from α-Syn, mainly via exosomes, activates microglia via the P2X7R/PI3K/AKT signalling pathway, subsequently mediates excessive microglial neurotoxicity and causes neuronal death. Decreasing the release of CTSL-containing exosomes substantially reduces the detrimental effects of activated microglia on neurons in a cellular model of PD.

## Results

### Extracellular α-Syn oligomers-induced microglial activation increases CTSL and exosome release

**Fig. 1:**
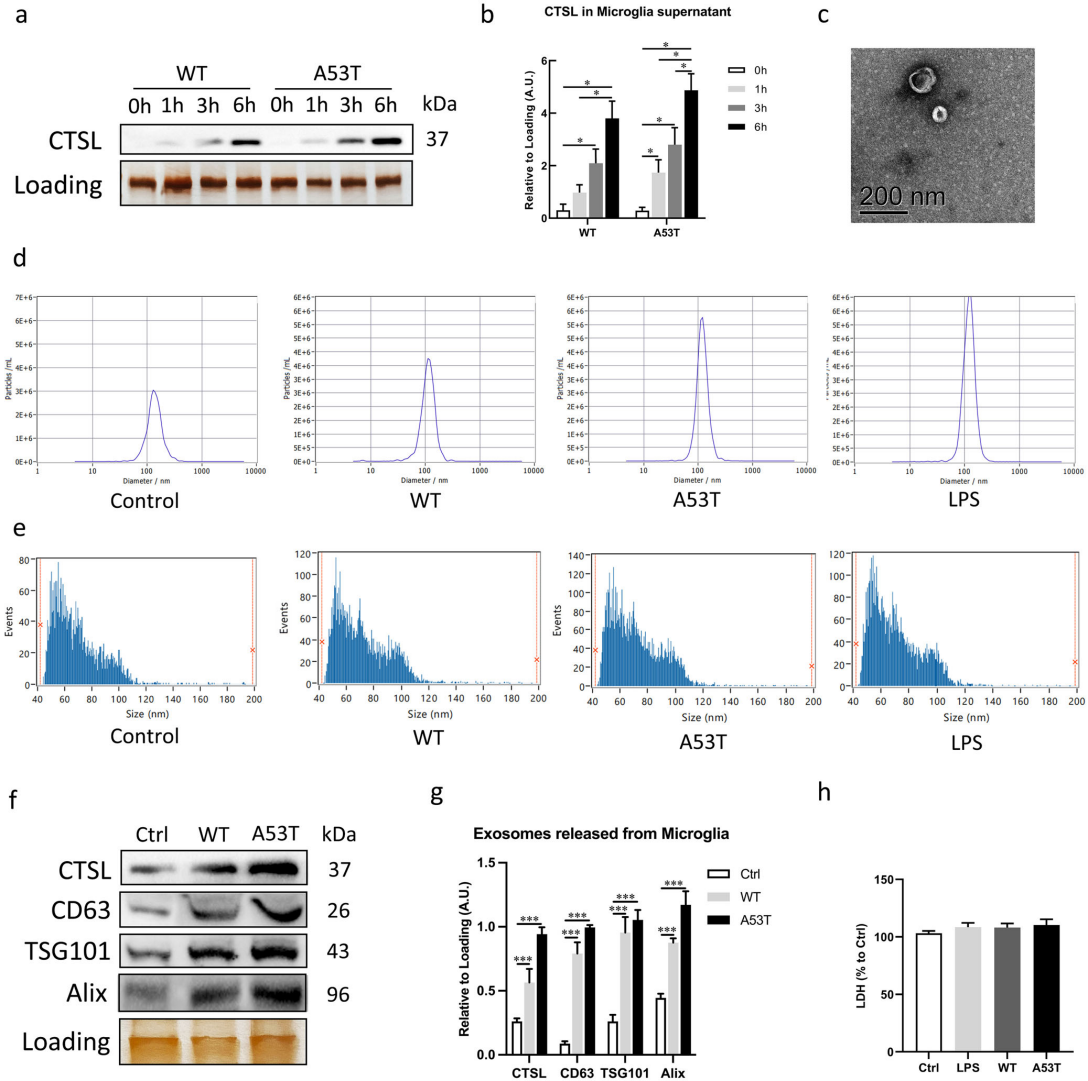
Extracellular α-Syn oligomers promote microglial CTSL and exosome release, and exosomes carry CTSL in α-Syn-activated microglia in a time-dependent manner. **a**, **b** Western blot and quantitative analysis of CTSL release from microglia stimulated with extracellular WT or A53T α-Syn oligomer for up to 6 h (*n* = 4); *p < 0.05. **c** Representative TEM images of exosomes isolated from the supernatant of primary microglia. Scale bar: 200 nm. **d**, **e** Analysis of the particle number and size of microglia-derived exosomes from the different treatment groups by NTA and a Flow Nano Analyser (*n* = 4). **f**, **g** The results of Western blot analysis showing the presence of the exosomal markers CD63, TSG101 and Alix, and a high level of CTSL in exosomes from WT and A53T α-Syn oligomer-activated microglia (*n* = 4); ***p < 0.001. **h** LDH levels in the microglial culture medium were measured by ELISA after different treatments (*n* = 6). Ctrl = untreated control. Error bars represent s.e.m.

As discussed earlier, CTSL is highly expressed in microglia and can be detected in the body fluids of PD patients^28,29^. To evaluate the effects of α-Syn oligomers-induced microglial activation on the release of CTSL, primary microglia were stimulated with 250 nM recombinant wild-type (WT) or A53T α-Syn oligomers. The volume of the supernatant normalised to the corresponding whole-cell protein concentration was used to reflect the amount of CTSL secreted from the same number of cells. Western blot analysis showed that both WT and A53T α-Syn oligomers promoted the release of CTSL from microglia into the supernatant in a time-dependent manner. CTSL was released after 1 h of stimulation with α-Syn oligomers, and the highest level of CTSL release was reached at 6 h, with the levels of CTSL being increased at earlier time points when A53T α-Syn oligomer was used as a stimulant (Fig. 1a, b). Notably, 100 ng/ml LPS was used as a positive control (Supplementary Fig. 1a, b). We further attempted to determine whether microglia can release a large number of exosomes after stimulation with α-Syn oligomers. Therefore, the exosome-rich fraction was isolated and purified from the microglia supernatant. TEM revealed that the exosomes exhibited a cup-shaped morphology (Fig. 1c). To quantitatively evaluate exosome release, three different exosome analysis techniques were used. First, the size and number of isolated exosomes were determined by NTA. Intriguingly, we demonstrated that many more exosomes were secreted from microglia treated with either form of α-Syn oligomers, especially those treated with α-A53T Syn, than from control microglia (Fig. 1d). Second, as expected, similar results, suggesting that α-Syn oligomers activation led to increased release of exosomes from microglia were obtained with the Flow Nano Analyser (Fig. 1e). The relevant quantitative data were showed in Supplementary Fig. 1c. Third, secreted exosomes isolated from the same number of cells were subjected to western blot analysis of specific exosome markers, including CD63, TSG101 and Alix. These three marker proteins were highly enriched in the WT and A53T α-Syn oligomers-treated microglia for 6 h compared with the untreated microglia; in addition, a high level of CTSL was also detected in the released exosomes isolated from the supernatant of α-Syn oligomers-stimulated microglia (Fig. 1f, g). Moreover, to eliminate the effect of α-Syn oligomer itself, we measured α-Syn levels in the exosome samples. The data in Supplementary Fig. 1d demonstrate that exosomes from microglia contained very little α-Syn protein. In addition, actin was only detectable in the cell lysates, while CD63 and TSG101 were also detectable in the cell lysates, demonstrating the absence of cellular components and other vesicles in the exosome preparations (Supplementary Fig. 1e). Furthermore, to eliminate the possibility that the increased number of exosomes observed in conditioned medium from microglia treated with LPS or α-Syn oligomers was due to disruption of the cell plasma membrane by these treatments, we measured LDH levels in the medium. The results indicated that no cell breakage was detected at the sublethal concentrations used (Fig. 1h). On the other hand, other cathepsins, such as Cathepsin B (CTSB), is also reported to be released via exosomes^30^, in consequence, to further confirm the specific pathogenic role of α-Syn oligomers-induced CTSL secretion from microglia in PD pathogenesis, we also detected the CTSB level in exosomes secreted by α-Syn oligomers-activated microglia. As shown in Supplementary Fig. 1f and g, stimulation with α-Syn oligomers did not result in more CTSB secretion through exosomes compared with controls. Meanwhile, non-pathogenic α-Syn monomers and γ-synuclein (γ-Syn), a kind of aggregate-prone proteins^31^, which has been reported to cause other degenerative diseases and brain tumours^32,33^, were also used to stimulate microglia under the same conditions. The results showed that neither α-Syn monomers nor γ-Syn aggregation induced significant release of CTSL-containing exosome compared with the control group (Supplementary Fig. 1h, i). There were no statistically significant differences in the above experimental results.

### CTSL release from α-Syn oligomers-activated microglia is mostly mediated by exosomes

**Fig. 2:**
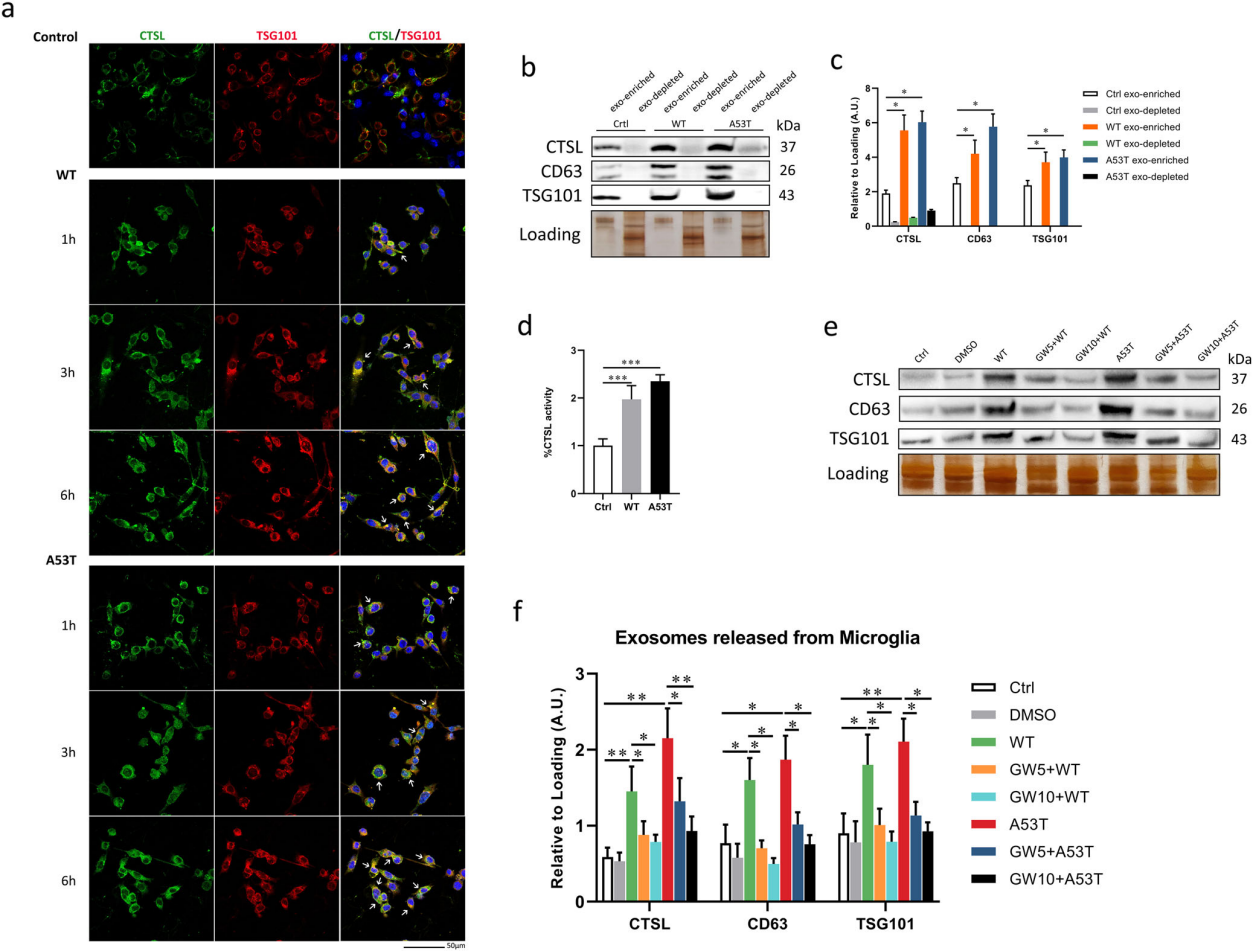
The main mechanism by which CTSL is released from α-Syn oligomers-activated is exosome-mediated release. **a** Representative confocal microscopy images of immunofluorescence staining for CTSL and the specific exosome marker TSG101. When primary microglia were activated by treatment with WT or A53T α-Syn oligomer for up to 6 h, the colocalization of CTSL and TSG101 (indicated by arrows) increased over time (*n* = 3). Scale bar: 50 μm. **b**, **c** The cell supernatant and secreted exosomes were isolated from the same number of microglia, and the protein levels of CTSL in both the exosome-enriched and exosome-depleted fractions were assayed by western blotting (*n* = 4); *p < 0.05. **d** Measurement of CTSL activity in exosome-enriched lysates from untreated and α-Syn oligomers-treated microglia. Exosome lysates without the addition of substrate were used as background controls (*n* = 6); ***p < 0.001. **e**, **f** Exosomes were isolated from the supernatants of control microglial and WT and A53T α-Syn oligomer-treated microglia in the presence and absence of GW4869. CTSL, CD63, and TSG101 levels in exosome lysates were measured by western blotting (*n* = 4); *p < 0.05, **p < 0.01. Ctrl = untreated control; exo = exosome; GW5 = 5 μM GW4869; GW10 = 10 μM GW4869. Error bars represent s.e.m.

To investigate whether CTSL is released via exosomes, microglia were treated with WT or A53T α-Syn oligomer, and then colocalization of CTSL with the exosomal marker TSG101 was assessed using immunofluorescence staining (Fig. 2a). The colocalization of CTSL and the exosome marker TSG101 was increased in a time-dependent manner and was most obvious for up to 6 h. Therefore, considering the results of immunofluorescence and western blot analysis, we chose 6 h as the treatment time point for all subsequent experiments. We measured CTSL levels in both the exosome-enriched and exosome-depleted supernatant fractions of α-Syn oligomers-treated and untreated microglia by western blot analysis. We observed that the majority of CTSL was concentrated in exosomes, while CTSL was almost undetectable or was only present at a very low level in the exosome-depleted fraction (Fig. 2b, c). Bovine serum albumin (BSA) protein was detected in only exosomes-depleted supernatants of each group as loading control (Supplementary Fig. 1j). We next assessed the activity of CTSL in exosomes, and similar experiments were performed. CTSL activity in the exosome-enriched fraction from each of the α-Syn oligomers-treated groups was significantly increased (Fig. 2d). Furthermore, to assess whether exosome-mediated release is the main mechanism of CTSL release from α-Syn oligomers-activated microglia, we treated the cultured cells with GW4869, a membrane neutral sphingomyelinase (nSMase) inhibitor, for 24 h prior to exosome isolation. GW4869 has been confirmed to markedly reduce exosome release, and preincubating cells with 0.5–20 µM GW4869 can prevent exosome production^34,35^. We used DMSO (the same volume of DMSO as that in 10 µM GW4869) as a control for GW4869 because GW4869 was dissolved in DMSO. Incubation with GW4869 not only strikingly reduced the levels of CD63 and TSG101 but also diminished the level of CTSL in exosomes isolated from α-Syn oligomers-activated microglia, confirming that exosomes carried CTSL as cargo (Fig. 2e, f). LPS (100 ng/ml) was used as a positive control (Supplementary Fig. 2a, b). Taken together, our results revealed that α-Syn oligomer increases CTSL release from microglia via exosomes.

### Extracellular α-Syn oligomers stimulate the release of CTSL-containing exosomes from microglia through P2X7R

**Fig. 3:**
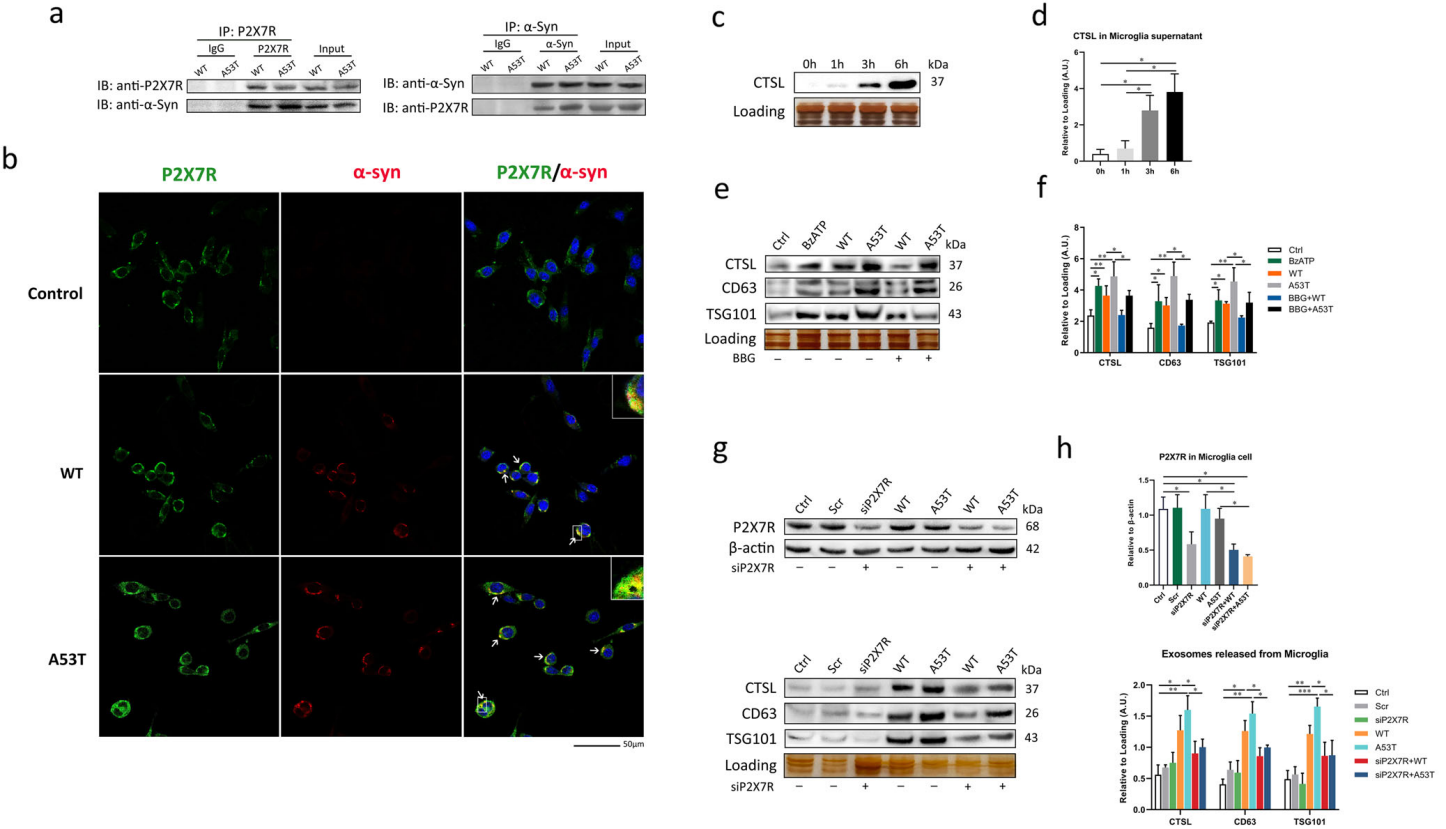
P2X7R is necessary for the release of CTSL-containing exosomes from α-Syn oligomers-treated microglia. **a** Extracellular α-Syn oligomer interacts with P2X7R. Western blot analysis of α-Syn expression in microglia after treatment with WT or A53T α-Syn oligomer for up to 6 h. WT and A53T α-Syn oligomer were coimmunoprecipitated with P2X7R, with A53T α-Syn oligomer showing a stronger interaction. **b** Representative confocal microscopy images of immunofluorescence staining for α-Syn (red) and P2X7R (green) after treatment with A53T or WT α-Syn oligomer for up to 6 h to observe colocalization (indicated by arrows). Scale bar: 50 μm. **c**, **d** Western blot and quantitative analysis of CTSL release from the supernatant of microglia stimulated with BzATP (100 μM), an agonist of P2X7R, for up to 6 h (*n* = 4); *p < 0.05. **e**, **f** BzATP obviously induced CTSL release from α-Syn oligomers-activated microglia via exosomes, while pretreatment with BBG (1 μM), an antagonist of P2X7R, effectively reversed CTSL-containing exosome release (*n* = 4); *p < 0.05, **p < 0.01. **g**, **h** Western blot and quantitative analysis of P2X7R expression in microglia transfected with nonspecific (scramble siRNA) (20 nM) or P2X7R siRNA (20 nM) to confirm P2X7R knockdown. Densitometric analysis of the number of CTSL-containing exosomes released from microglia transfected with scramble or P2X7R siRNA after treatment with WT or A53T α-Syn oligomer for 6 h (*n* = 4); *p < 0.05, **p < 0.01, ***p < 0.001. Ctr = untreated control; Scr = scramble; siP2X7R = P2X7R siRNA. Error bars represent s.e.m.

As discussed earlier, in our previous experiments, we demonstrated that α-Syn oligomers interacts with P2X7R in BV2 cells^4^. Here, co-IP and western blotting were used to confirm that this phenomenon occurs in microglia (Fig. 3a), meanwhile, immunostaining using antibodies against α-Syn oligomers and P2X7R for up to 6 h also showed the interaction between the two proteins (Fig. 3b). In co-IP experiment, A53T α-Syn oligomer interacted more strongly with P2X7R than WT α-Syn oligomer. The α-Syn oligomer, rather than the monomer form, has long been identified as the pathogenic protein of PD^36^. As an additional control experiment, similar coimmunoprecipitation experiments were performed on the α-Syn monomers. As shown in Supplementary Fig. 2d, neither WT α-Syn monomer nor A53T α-Syn monomer interacted with P2X7R. These observations suggest that α-Syn oligomers may induce CTSL-containing exosome release by interacting with P2X7R. To test this hypothesis, we first treated microglia with 100 mM 2’(3’)-O-(4-benzoylbenzoyl) adenosine 5’-triphosphate (BzATP), a P2X7R agonist that is 10-fold more potent than ATP^37^, with 250 nM BSA being used as a negative control Supplementary Fig. 2c). Our results showed that the highest level of CTSL release from microglia occurred approximately 6 h after BzATP treatment (Fig. 3c, d) and that the secretion of CTSL occurred mainly through exosomes (Fig. 3e, f). Moreover, incubation with Brilliant Blue G (BBG), a P2X7R antagonist, 10 min before α-Syn oligomers treatment significantly reduced the secretion of CTSL via exosomes in both the WT and A53T α-Syn oligomers-treated groups (Fig. 3e, f), implying the involvement of P2X7R activation in this process. Similar results were obtained when P2X7R was knocked down in microglia before treatment with α-Syn oligomers (Fig. 3g, h). These investigations provide evidence that extracellular α-Syn oligomers can directly interact with and activate P2X7R and trigger CTSL release from microglia through exosomes.

### α-Syn oligomers activate CTSL-containing exosome release from microglia via the P2X7R/PI3K/AKT signalling pathway

**Fig. 4:**
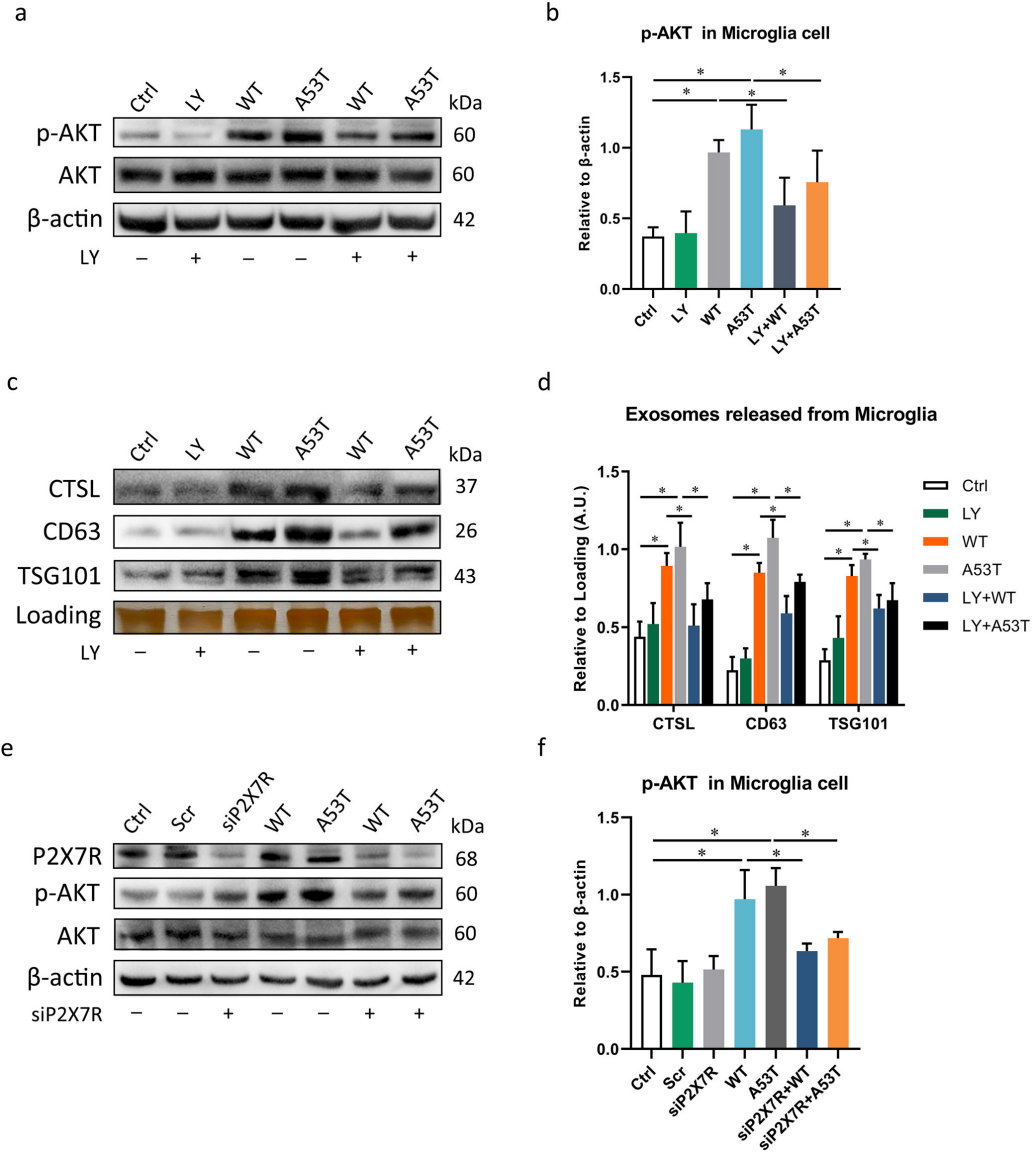
P2X7R stimulates PI3K/AKT signalling in microglia in response to extracellular α-Syn oligomers, and α-Syn oligomers activate microglial CTSL-containing exosome release via the P2X7R/PI3K/AKT signalling pathway. **a**, **b** p-AKT levels in microglia were increased after treatment with WT or A53T α-Syn oligomer, with A53T α-Syn oligomer causing a more significant increase in AKT phosphorylation. Inhibition of PI3K by LY294002 (50 μM) blocked α-Syn-induced phosphorylation of AKT (*n* = 4); *p < 0.05. **c**, **d** Exosomes were isolated from the supernatants of control and α-Syn oligomer-treated microglia in the presence or absence of LY294002. CTSL, CD63, and TSG101 levels in exosome lysates were quantitatively analysed by western blotting (*n* = 4); *p < 0.05. **e**, **f** p-AKT expression in microglia transfected with scramble or P2X7R siRNA after stimulation with WT or A53T α-Syn oligomer. Knockdown of P2X7R rescued the increase in p-AKT expression in BV2 cells in response to stimulation with WT or A53T α-Syn oligomer, implying that α-Syn oligomer-mediated stimulation of P2X7R is an upstream event that activates PI3K/AKT signalling and ultimately CTSL-containing exosome release (*n* = 4); *p < 0.05. Ctrl = untreated control; LY = LY294002; Scr = scramble; siP2X7R = P2X7R siRNA. Error bars represent s.e.m.

To identify the specific signalling pathways downstream of P2X7R, we next assessed the potential involvement of the PI3K/AKT pathway in α-Syn oligomers-induced CTSL release. Aberrant signalling through the PI3K/AKT pathway has been implicated in both PD^38^ and the expression and secretion of CTSL^15^. To determine the role of PI3K/AKT signalling in promoting CTSL-containing exosome release, the level of phospho-AKT (p-AKT) was measured in the WT and A53T α-Syn oligomers-treated groups and the control group. Stimulation of microglia with WT or A53T α-Syn oligomer resulted in an increase in p-AKT expression, and when microglia were pretreated with 2-(4-morpholinyl)-8-phenyl-4H-1-benzopyran-4-one (LY294002), a potent and specific cell-permeable inhibitor of PI3K, phosphorylation of AKT was effectively suppressed (Fig. 4a, b). Moreover, pretreatment with LY294002 markedly decreased the secretion of CTSL via exosomes (Fig. 4c, d). Our results have thus far revealed that α-Syn oligomers promote CTSL-containing exosome release in a manner that is dependent upon PI3K/AKT signalling.

We next analysed the expression of p-AKT after P2X7R was knocked down to identify whether P2X7R is upstream of the PI3K/AKT signalling pathway. As expected, knockdown of P2X7R rescued the increase in p-AKT expression in microglia in the WT and A53T α-Syn oligomers-treated groups compared with the control and scramble siRNA-treated groups (Fig. 4e, f). Collectively, these results suggest that the P2X7R/PI3K/AKT signalling pathway is strongly linked to CTSL-containing exosome release from α-Syn oligomers-treated microglia.

### α-Syn oligomers-treated microglia exert neurotoxic effects through the release of CTSL-containing exosomes

**Fig. 5:**
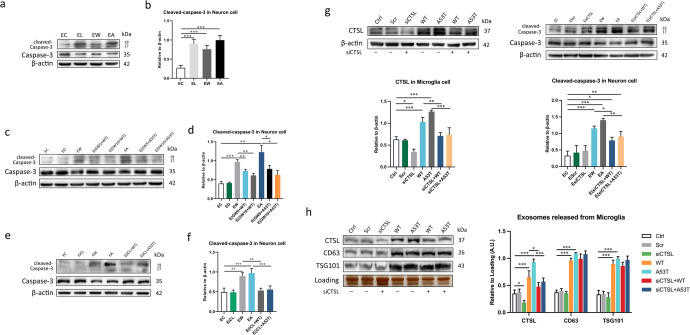
CTSL-containing exosomes released from activated microglia promote apoptosis of neurons. **a**, **b** Primary cortical neurons were treated with exosomes isolated from microglia treated with or without WT or A53T α-Syn oligomer. Western blot and quantitative analysis of cleaved-caspase-3 expression in neurons treated for 24 h with exosomes from untreated microglia or microglia treated with LPS or WT or A53T α-Syn oligomer (*n* = 3); ***p < 0.001. **c**, **d** The level neuronal apoptosis was determined after co-culture with exosomes from microglia pretreated with or without GW4869 (*n* = 3); *p < 0.05, **p < 0.01, ***p < 0.001. **e**, **f** The level of apoptosis of neurons cocultured with exosomes from microglia treated with or without iCL (n = 3); **p < 0.01, ***p < 0.001. **g** Western blot and quantitative analysis of CTSL expression in microglia transfected with nonspecific (scramble siRNA) (20 nM) or P2X7R siRNA (20 nM) to confirm CTSL knockdown. Exosomes secreted by α-Syn oligomers-activated CTSL knockdown microglia induce less neuronal death (*n* = 3). **h** Densitometric analysis of the number of CTSL-containing exosomes released from microglia transfected with scramble or CTSL siRNA after treatment with WT or A53T α-Syn oligomer for 6 h (*n* = 3); *p < 0.05, **p < 0.01, ***p < 0.001. The amount of exosomes used in each group was secreted by approximately 3*10^7^ microglia cells. Ctrl = untreated control; Scr = scramble; siCTSL = CTSL siRNA; EC = exosomes from untreated microglia (the control group); EL = exosomes from LPS-treated microglia; EW = exosomes from WT α-Syn oligomer-treated microglia; EA = exosomes from A53T α-Syn oligomer-treated microglia; ED = exosomes from DMSO-treated microglia; EiCL = exosomes from iCL-treated microglia; GW5 = 5 μM GW4869; GW10 = 10 μM GW4869; EsiCTSL = exosomes from CTSL knockdown microglia. Error bars represent s.e.m.

**Fig. 6:**
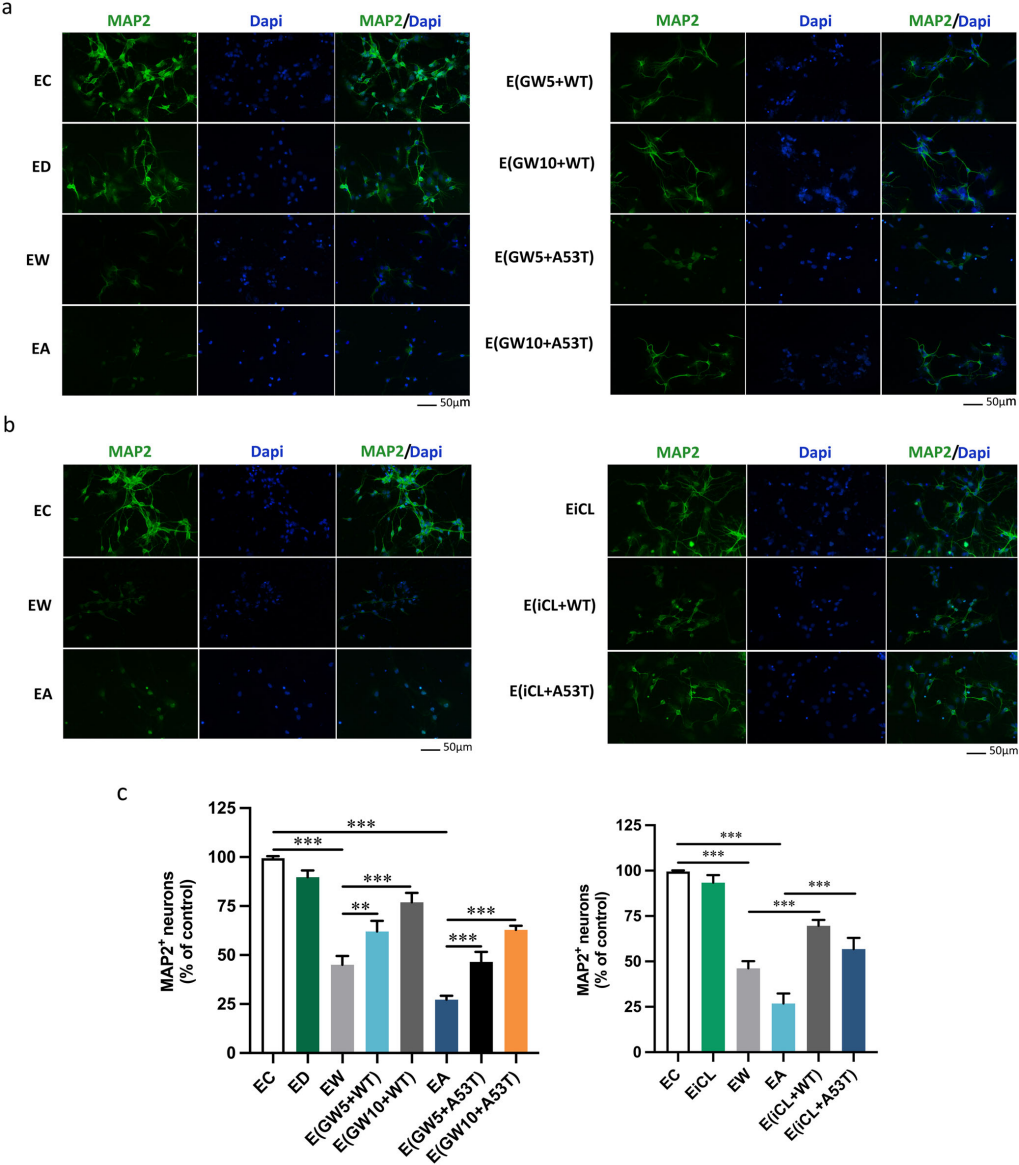
Representative immunofluorescence images of neurons labelled with MAP2 illustrating the neurotoxic potential of CTSL-containing exosomes. **a**, **b** Treatment with GW4869 or the CTSL inhibitor iCL (20 μM) prior to exosome isolation rescued CTSL-containing exosome-induced neurotoxicity in a dose-dependent manner in each experimental group, as determined by MAP2 immunostaining, suggesting a direct neurotoxic role for CTSL released from α-Syn oligomers-treated microglia. Scale bar: 50 μm. **c** Quantification of the percentage of MAP2^+^ neurons for the experiments depicted in panels **a** (up) and **b** (down). Results are representative of three fluorescent images of each group from three independent experiments. The amount of exosomes used in each group was secreted by approximately 10^4^ microglia cells. EC = exosomes from untreated microglia (the control group); EW = exosomes from WT α-Syn oligomer-treated microglia; EA = exosomes from A53T α-Syn oligomer-treated microglia; ED = exosomes from DMSO-treated microglia; EiCL = exosomes from iCL-treated microglia; GW5 = 5 μM GW4869; GW10 = 10 μM GW4869. Error bars represent s.e.m.

Excessive activation of microglia, which involves the release of exosomes, is believed to contribute to neuronal death in neurodegenerative diseases, and mechanisms by which the CNS is damaged are very complicated and involve much more than inflammation and oxidative stress^39^. To investigate the adverse impact of CTSL secreted from α-Syn oligomers-treated microglia on neurons, exosomes isolated from the microglial supernatant were resuspended in neurobasal medium and then cocultured with neurons. The volume of culture medium used to resuspend the exosomes was adjusted based on the exosomal protein concentration from microglia to ensure that the concentration of exosomes was consistent per unit volume of resuspending, therefore the same amount of exosomes could be used for co-culture with each group of neurons in subsequent experiments. To explore the effect of CTSL-containing exosomes released from microglia on neuronal apoptosis, we initially assessed neurotoxicity by quantifying the expression of the apoptotic protein caspase-3 by western blotting (Fig. 5a–f) and the neuronal marked MAP2 by immunostaining (Fig. 6). Similar to prior experiments, we used LPS as a positive control (Fig. 5a, b), and the same volume of DMSO administered to the GW4869 treatment groups was used as a negative control. The neurotoxic effect of CTSL-containing exosomes released from activated microglia was mostly blocked by increasing concentrations of GW4869 (Fig. 5c, d). Consistently, treatment with GW4869 prior to exosome isolation rescued exosome-induced neurotoxicity in a dose-dependent manner, as shown by MAP2 immunostaining (Fig. 6), illustrating the obvious role of exosomes in the neurotoxicity of α-Syn oligomers-treated microglia. Furthermore, microglia were preincubated with or without the CTSL inhibitor NapSul-Ile-Trp-CHO (iCL) (20 mM) for 12 h before α-Syn oligomers stimulation. As expected, pretreatment with iCL significantly ameliorated neuronal apoptosis, verifying that inhibition of CTSL directly rescued neuronal death (Figs. 5e, f and 6). In addition, to strengthen this conclusion, similar treatments were performed in the setting of CTSL knockdown in microglia. Cleaved-caspase-3 was sharply attenuated, but not completely inhibited, in neuron cells treated with conditioned media from α-Syn oligomers-activated microglia transfected with CTSL siRNA (Fig. 5g). Furthermore the expression levels of CTSL in WT and A53T α-Syn oligomers-activated microglia were upregulated, and correspondingly, after CTSL was knocked down, the level of CTSL in exosomes secreted by α-Syn oligomers-activated microglia reduced significantly, but the amount of exosomes itself did not decrease (Fig. 5h). Our results confirmed α-Syn oligomers not only regulate the release of CTSL-containing exosomes by activating the P2X7R/PI3K/AKT signalling pathway, but also regulate the expression level of CTSL, making it play a crucial role in causing neuronal death.

### Exosomes mediate extracellular transmission of CTSL between microglia and neurons

**Fig. 7:**
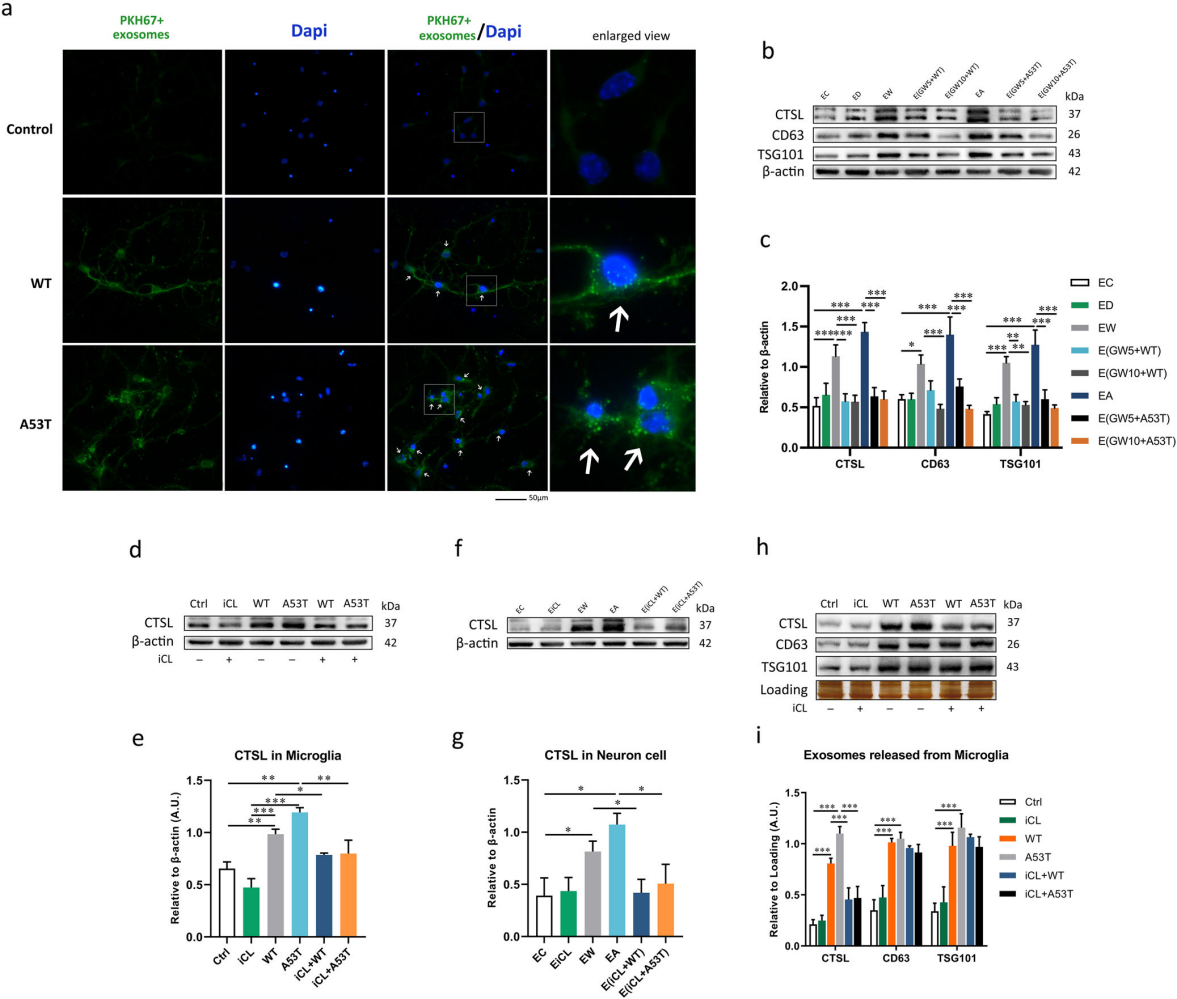
Secretion of exosomes by microglia is responsible for the transmission of CTSL to neurons. **a** Exosomes isolated from the same volume of culture medium from microglia exposed to different experimental conditions were labelled with the dye PKH67. These exosomes were then added to recipient neurons and incubated for 1 day. The neurons were then fixed with 4% PFA (*n* = 3). Exosomes secreted by α-Syn oligomers-treated microglia were endocytosed by neurons (spot-shaped, indicated by arrows). The amount of exosomes used in each group was secreted by approximately 10^4^ microglia cells. Scale bar: 50 μm. **b**, **c** The level of CTSL, CD63 and TSG101 in neurons cocultured with exosomes from microglia pretreated with or without GW4869 was measured by western blotting and quantitatively analysed (*n* = 3); *p < 0.05, **p < 0.01. GW4869 treatment reduced exosome internalisation by neurons, resulting in lowers CTSL expression. **d**, **e** Pretreatment with the CTSL inhibitor iCL decreased the expression of CTSL in microglia, as determined by western blotting and quantitative analysis (*n* = 3); *p < 0.05, **p < 0.01, ***p < 0.001. **f**, **g** Due to the inhibitory effect of iCL, less CTSL was released by α-Syn-activated microglia and internalised by neurons (*n* = 3); *p < 0.05. **h**, **i** CTSL, CD63, and TSG101 expression in exosome lysates from microglia was measured by western blotting. Administration of iCL prior to activation with α-Syn did not affect the reduction in exosome release from microglia, which once again proves that CTSL, not exosomes, is responsible for neuronal damage (*n* = 3); ***p < 0.001. The amount of exosomes used in each group was secreted by approximately 3*10^7^ microglia cells. EC = exosomes from untreated microglia (the control group); EW = exosomes from WT α-Syn-treated microglia; EA = exosomes from A53T α-Syn-treated microglia; ED = exosomes from DMSO-treated microglia; EiCL = exosomes from iCL-treated microglia; Ctrl = untreated control; GW5 = 5 μM GW4869; GW10 = 10 μM GW4869. Error bars represent s.e.m.

As shown by the results described above, exosomes obtained from α-Syn oligomers-treated microglia can carry CTSL. Exosomes can fuse with the plasma membrane of recipient cells, releasing cargo directly into the cytosol, or can be internalised by endocytosis^40^. To determine whether CTSL-containing exosome uptake occurred after neurons were cocultured with exosomes secreted by microglia, we labelled purified exosomes with the membrane dye PKH67 and then added these PKH67^+^ exosomes to primary neurons. The results showed that after α-Syn oligomers treatment, the internalisation of exosomes by neurons was obviously increased (Fig. 7a). Subsequently, we assessed the expression of CTSL in neurons cocultured with exosomes collected from microglia-conditioned medium by western blot analysis. Co-culture with exosomes from microglia stimulated with WT or A53T α-Syn oligomer resulted in an obvious increase in CTSL levels in neurons, as well as the amount of exosomes (Fig. 7b, c). It seemed that exosomes facilitated transmission of CTSL over a long distance and were eventually endocytosed by neurons. Interestingly, after microglia were treated with GW4869 for 24 h and then treated with WT or A53T α-Syn oligomer, GW4869 markedly reduced the internalisation of CTSL-containing exosomes by neurons (Fig. 7b, c). These results suggest that CTSL-containing exosomes were able to infect heathy neurons and that reducing the release of CTSL-containing exosomes from microglia could reduce the transmission of CTSL to neurons.

We next assessed the CTSL level in neurons cocultured with conditioned medium from microglia preincubated with or without the CTSL inhibitor iCL. Results showed that iCL pretreatment reduced the expression of microglial CTSL (Fig. 7d, e), and as a consequence, iCL pretreatment led to a decrease in the secretion of CTSL through exosomes and reduced internalisation of CTSL by neurons (Fig. 7f–i). There was no significant decrease in the amount of exosome release in the iCL pretreatment groups (Fig. 7h, i). Again, these results provide strong evidence that CTSL in exosomes is the main culprit of microglia-mediated neuronal toxicity.

## Discussion

Our previous studies identified extracellular α-Syn oligomers as a potential inducer of microglial activation, which significantly contributes to neuronal damage and thus accelerates the progression of PD. However, the key molecular mechanisms remain unknown. Combining proteomics technology and molecular analyses, our present study clearly reveals that CTSL-containing exosomes exert a neurotoxic effect during excessive microglial activation. First, using a well-characterised human recombinant α-Syn oligomers-induced microglial activation model, we showed that exosomes released from these microglia contained CTSL, which is a key enzyme involved in the pathogenesis of PD, and that CTSL was released from α-Syn oligomers-treated microglia into the extracellular fluid primarily via exosomes. Second, we demonstrated that BzATP, a classic agonist of P2X7R, obviously increased the number of CTSL-containing exosomes released from microglia, which was decreased in the P2X7R antagonist pretreatment groups and P2X7R knockdown groups. Interestingly, activation of PI3K/AKT signalling downstream of P2X7R was confirmed to affect the release of CTSL-containing exosomes, while an inhibitor of PI3K suppressed α-Syn oligomers-induced microglial activation and reduced the release of exosomes and CTSL. Third, activation of P2X7R/PI3K/AKT signalling was required for the neurotoxic effect of CTSL-containing exosomes from α-Syn oligomers-induced microglia, resulting in the death of primary neurons. Finally, we used PKH67 to label exosomes and investigated the effects of microglia on CTSL transmission in the brain. Based on these observations, we propose that CTSL is released from activated microglia via exosomes through the P2X7R/PI3K/AKT signalling pathway and that exosomal CTSL may be used as a potential biomarker of PD for early diagnosis and prognosis.

Uncontrolled microglial activation, which is a critical contributor to the pathogenesis of PD, is an inevitable and major response to CNS insult^41,42^. Indeed, activated microglia secrete the lysosome protease CTSL^6,28,43^, and CTSL has been found to be involved in some in vitro and in vivo models of several neurodegenerative diseases, including PD^44^; however, few studies have explored the specific mechanism of CTSL secretion by microglia and clarified whether secreted CTSL contributes to the pathogenesis of PD. Our current research exactly fills this gap, as our data not only provide solid evidence for the role of CTSL as a pathogenic protein that causes neuronal death in PD but also prove that exosome-mediated release is the main mechanism by which CTSL is released from the cytosol of α-Syn oligomers-treated microglia into the extracellular compartment. Previous studies have suggested that extracellular vesicles (EVs), including exosome-like vesicles, are released from specialised cells and contain the protease CTSL and many immunomodulatory molecules that can be delivered to host cells^45,46^. Consistent with these results, by using a well-established cellular model of PD, we observed that α-Syn oligomers-induced microglial activation led to marked release of CTSL into the cell supernatant in a time-dependent manner. In addition, administration of a classical inhibitor of exosomes, GW4869, resulted in a dose-dependent decrease in the levels of both CTSL and exosome. Therefore, we concluded that exosomes are the primary facilitators of CTSL release by α-Syn oligomers-treated microglia.

Previous research revealed that activation of P2X7R can regulate the secretion of exosomes by microglia^47^. We proposed that P2X7R is a key contributor to α-Syn oligomers-mediated microglial activation in a previous study, and the obvious and strong interaction between α-Syn oligomers and P2X7R was verified in the current research. We next explored the potential involvement of P2X7R in the release of CTSL-containing exosomes from microglia induced by extracellular α-Syn oligomers, given that this receptor has been reported to be closely associated with cytokine-independent release of CTSL from macrophages in patients with rheumatoid arthritis^48^ and that ATP-induced CTSL release is abolished by P2X7R antagonists and in P2X7R^−/−^ mice^13^. Our findings demonstrated that P2X7R is a key contributor to CTSL-containing exosome release. Furthermore, we showed that the P2X7R agonist BzATP significantly promoted the secretion of CTSL and that P2X7R knockdown and BBG administration prevented the release of most exosomes and the CTSL they carried. In addition, we confirmed that CTSL is transported between microglia and neurons, as CTSL-containing exosomes were endocytosed by neurons. It has been reported that exosomes transport to distal sites and internalised into neighbouring target cells through endocytosis or membrane fusion^49^, Consequently, cell-to-cell transmission of CTSL could be one of the major mechanisms of PD progression and deterioration. This process could be mediated by several mechanisms of cellular release and uptake, and the mechanism of CTSL uptake will be the focus of our future research. Notably, we observed an obvious decrease in both the number of exosomes and the amount of CTSL secreted by α-Syn oligomers-stimulated microglia after BBG pretreatment; however, secretion was not completely blocked. This phenomenon can be explained by the activation of surface receptors that also promote exosome secretion other than P2X7R on microglia. Exosome secretion has no specificity, but according to the results we described above, the release of CTSL, one cargo of exosomes, by α-Syn oligomers-activated microglia through P2X7R is specific to some extent, and other signalling pathways might participate in this process.

Another important observation of this study is that the neurotoxicity of α-Syn oligomers-treated microglia was almost abolished by pretreatment with GW4869, which indicates that CTSL-containing exosomes are the neurotoxic factors in α-Syn oligomers-treated microglia. Next, we use iCL, an inhibitor of CTSL. Strikingly, iCL treatment reduced damage to neurons, which indicates that CTSL-carrying exosomes from microglia are sufficient to induce the major pathological features of PD. As far as we know, there are no specific studies on the mechanism of iCL. In our study, the binding place of iCL and CTSL is not clear (cell membrane or lysosomal, etc.), thus this is also what we will further explore in future experiments.

In the above results we have established that α-Syn oligomers-stimulation did not cause microglia to secrete CTSB through exosomes. Next, we found that knockdown of the CTSL reduced the CTSL-secreted capacity of α-Syn oligomers-activated microglia and partially alleviated the neurotoxicity of CTSL-containing exosomes on neurons. With further analysis, we observed the level of CTSL in α-Syn oligomers-treated microglia increased obviously, leading to the increase of CTSL secretion in exosomes (Figs. 5g and 7d, e). To summarise, CTSL is a key component involved in α-Syn oligomers-mediated microglial damage to neurons. Previous studies have confirmed that microglia are actively involved in the process of cell-to-cell transmission of proteins through the release of exosomes^50^, which is consistent with our findings that the microglia-to-neuron transmission of CTSL-containing exosomes leads to a vicious neurotoxic cycle. We observed that PKH67 labelled exosomes secreted by microglia were endocytosed by neurons. Meanwhile, the expression of exosomal markers in cell lysates sample of neurons also increased evidently (Fig. 7b). Notably a significant increase in neuronal apoptosis and a decrease in MAP2 expression, which regulates neuronal development and structural stability. Taken together, these results suggest that inhibiting the release of CTSL-containing exosomes might be a neoteric therapeutic approach for the treatment of PD in patients. Even more importantly, because CTSL is abundantly expressed in both the CNS and peripheral blood^51,52^, qualitative or quantitative changes in exosomal CTSL levels may be a new and accessible biomarker for the early diagnosis of PD.

A consistent observation throughout this investigation was that regardless of time or quantity, the A53T mutant is much more potent than WT α-Syn oligomer in activating the release of CTSL-containing exosomes from microglia. One possible reason for this is that there are structural differences between the two oligomers, and A53T mutation has been shown to be more neurotoxic and more prone to aggregate into oligomers^53,54^, resulting in more sustained microglia activation^55,56^. Although the molecular mechanisms underlying α-Syn aggregation remain unknown, the A53T mutant has bias for more open structures, with the potential of binding more biological macromolecule like proteins^54^. This view is consistent with our findings and suggest another possible reason that there is a stronger interaction between A53T α-Syn oligomer and P2X7R (as demonstrated in Fig. 3), which leads to an enhanced downstream effect. Alternatively, the A53T mutant may provoke the release of CTSL through another signalling pathway besides the PI3K/AKT pathway, which merits further investigation in the future. Given these original findings, the domains that mediate the interaction between α-Syn oligomer and P2X7R will be explored in subsequent studies, and meanwhile we will prove this mechanism in PD patients, which may be a promising strategy for innovative treatment of PD.

In summary, this study identifies CTSL-containing exosome as a toxic mediator in synapse loss and neuron death in α-Syn oligomers-induced microglial activation via P2X7R/PI3K/AKT signalling pathway. Based on the above findings, using a siRNA specific for P2X7R or an inhibitor of P2X7R/PI3K reverses neurotoxicity by attenuating the release of the exosomal CTSL. Our data highlight the potential cellular mechanisms by targeting P2X7R, the key membrane protein of α-Syn oligomers-activated microglia and P2X7R-mediated secretory pathway of exosomes is responsible for the progressive spread of the microglial neurotoxicity and cell death in neighbouring neurons. We report that microglia are actively involved in the process of cell-to-cell transmission of CTSL through the release of exosomes, and also propose a new avenue for neuroprotective intervention strategies of PD by blocking the secretion of CTSL-containing exosomes. To manipulate this specific pathway, at least in theory, could be an effective way to treat PD patients.

## Methods

### Reagents and antibodies

Anti CTSL, anti Alix and anti P2X7R were purchased from Santa Cruz Biotechnology. Anti TSG101, anti CD63, anti α-synuclein and anti β-actin were purchased from Sigma-Aldrich (USA, St. Louis, Missouri). Anti AKT, anti p-AKT, anti caspase-3 and anti MAP2 were purchased from Cell Signalling Technology (USA). Anti CD11b and anti BSA were purchased from Abcam (UK). Anti CTSB was purchased from Proteintech (Wuhan, China). Purified human recombinant γ-Syn was purchased from absin (Shanghai, China) and endotoxin content of peptide was <0.1 ng/μg (1 IEU/μg) as determined by limulus amebocyte lysate (LAL) test determined at absin.

Lipopolysaccharides (LPS) from *Escherichia coli* 026:B6 were purchased from Sigma-Aldrich (USA, St. Louis, Missouri) and dissolved in H_2_O to create a 20 ng/μl of stock solution. LY294002 was purchased from Thermo Fisher (UK) and dissolved in dimethyl sulfoxide to create a stock solution of 25 mg/ml. BzATP, BBG and GW4869 were purchased from Sigma-Aldrich (USA, St. Louis, Missouri).

iCL was purchased from Enzo (USA) and it is a biomimetic material developed and synthesised based on E-64 and leupeptin, two natural inhibitors of CTSL. Studies have confirmed that CTSL and iCL have better adaptation in spatial structure, higher affinity, more specific binding and lower toxicity than natural inhibitors as well, and are widely used in vivo experiments in cells and animals^57,58^.

### Animals

C57BL/6 mice purchased from Shanghai SLAC Laboratory Animal Co., Ltd. (Shanghai, China) were used in this study. They were maintained on a 12-h light/dark cycle under standard conditions at a constant temperature (22 °C ± 2 °C) and humidity (50%–65%). Food and water provided ad libitum. All procedures involving animals were approved by the Shanghai Jiao Tong University School of Medicine Animal Care and Use Committee and conducted in accordance with the National Institutes of Health Guide for the Care and Use of Laboratory Animals (NIH Publications No. 8023, revised 1978).

### Primary cell culture

Primary cortical neurons were prepared from C57BL/6 mouse embryos on embryonic day 16–18 (E16-18). Pregnant mice were sacrificed under anaesthesia with sevoflurane, their embryos were recovered, and cerebral cortex tissues were collected from the embryonic brains. For neuron-enrichment, the meninges and blood vessels were carefully removed. The cerebral cortex tissues of the foetuses were removed, dissociated by mild trypsinization, and mechanically triturated in DNase solution (0.004% *w*/*v*) containing a soybean trypsin inhibitor (0.05% *w*/*v*). This mixture was centrifuged for 3 min at 1000 rpm, the supernatant was removed and the cell pellet was dissolved in neurobasal medium supplemented with 1% B-27 supplement (Thermo Fisher, UK), 5 mM l-glutamine, and 1% penicillin/streptomycin (PS; Thermo Fisher, UK). The cell suspension was seeded in 10 cm dishes and 12-well plates precoated with poly-d-lysine, and the plates were cultured in 95% air and 5% CO_2_ at 37 °C. The culture medium was replaced every 3−4 days. Neurons were used for experiments after 7 to 10 days in culture.

Primary mouse microglia were prepared from newborn C57BL/6 pups (1 day old) as described in a previous article^4^. Briefly, a total of 2 × 10^7^–2.3 × 10^7^ cells dissociated cells isolated from pooled neocortical tissues from male pups were plated in a 175 cm^2^ flask coated with poly-l-ornithine (Sigma-Aldrich, USA). After approximately 12–14 days in vitro, the flasks were shaken vigorously, and the medium was collected and centrifuged at 1000 rpm for 5 min to obtain a microglial pellet. For obtain purified exosomes from the cell culture supernatant, primary microglia were resuspended in DMEM/F-12 supplemented with 10% exosome-depleted FBS (SBI, USA) and 50 U/ml PS and separately seeded on poly-l-ornithine-coated coverslips in 12-well plates and 10 cm dishes for follow-up experiments. The cell purity was confirmed to be >98% by immunofluorescence using CD11b as a marker of microglia (Supplementary Fig. 2e).

To establish our cell model, purified human recombinant WT and A53T α-Syn (rPeptide, Athens, USA) were dissolved in ddH_2_O to create a 1 mg/ml stock solution and incubated at 37 °C in 5% CO_2_ for 7 days with constant agitation by using a mini Teflon stir bar to yield oligomeric α-Syn according to previous literatures^59–61^. Since potential contamination of endotoxin may confound the results of α-Syn on microglial activation, the amount of endotoxin in the purified α-Syn was determined at r-Peptide. The result showed that the content of endotoxin was <1.3 U/mg of peptide, which was consistent with previous studies and incapable of producing any significant neurotoxicity^59,62^.

### Characterisation of purified α-synuclein aggregation

The procedures for protein oligomers negative-staining were established by TEM following our previously described methods with little modification^63,64^. Briefly, 5–10 μl of each sample was dropped on a carbon support membrane, followed by dropwise addition of 5–10 μl of 1% uranyl acetate. Then the membrane was dried at room temperature for testing. TEM images were collected with a Hitachi HT7700 by Shanghai Yuyi Technology Co., Ltd (Supplementary Fig. 2f).

### Cell stimulation

Primary microglia were treated with LPS (100 ng/ml, E. coli, serotype 055: B5, Sigma-Aldrich, USA) or recombinant α-Syn oligomers (250 nM). Cells, cell supernatants and exosomes were collected for analysis of protein expression and other parameters (see below).

### Isolation and purification of exosomes

The supernatants of primary microglia collected from each sample were centrifuged at 2000 × *g* for 30 min to remove cell debris and dead cells (Beckman Coulter, Allegra X-14R). Exosomes were then purified from the supernatants using an exosome isolation kit (Thermo Fisher, UK, Cat^#^ 4478359). We added 0.5 volumes of the Total Exosome Isolation reagent into the required volume of cell-free culture media and then mixed the culture media/reagent mixture well by vortexing. After incubation at 4 °C overnight, samples were centrifuged at 10,000 × *g* for 1.5 h at 4 °C. The supernatant was aspirated and concentrated by a Macrosep Advance centrifugal device with a molecular weight cutoff of 1 kDa (Pall Life Sciences, MI) to prepare the exosome-depleted fraction, and the pellet containing exosomes at the bottom of the tube was resuspended in 150 μl RIPA buffer (Beyotime Biotechnology, China) to prepare the exosome-enriched fraction.

### Exosome identification

A total of 5 μl exosome suspension was spread on a copper grid at room temperature for 1 min. Filter paper was then used to remove the superfluous liquid. Afterwards, the exosomes were negatively stained using 1% (w/v) sodium phosphotungstate solution at room temperature for 3 min, and the solution was removed with filter paper. Then, the copper grid was placed under a tungsten lamp for 10 min. For transmission electron microscopy (TEM) (De Jong Instruments, USA), isolated and purified exosomes were fixed in 2% glutaraldehyde and 2% paraformaldehyde, dried, mounted and coated with gold/palladium. A Flow Nano Analyser (NanoFCM Co., Ltd, China) was used to determine the diameter and size distribution of the exosomes.

### Nanoparticle tracking analysis (NTA)

Exosome size and concentration were measured by using NTA with a ZetaView PMX 110 analyser (Particle Metrix, Meerbusch, Germany) and ZetaView 8.04.02 software. Briefly, isolated exosome samples were appropriately diluted using 1x PBS buffer (Biological Industries, Israel) to measure the particle size and concentration. NTA was performed at 11 positions. The ZetaView system was calibrated using 110 nm polystyrene particles. The temperature was maintained at approximately 25 °C to 26 °C.

### PKH67 labelling of exosomes and lactate dehydrogenase (LDH) assay

Purified exosomes were labelled with a PKH67 green fluorescent labelling kit (Sigma-Aldrich, USA) according to the manufacturer’s instructions. Briefly, exosome pellets obtained from 20 ml culture medium were resuspended in 500 ml Dilution C, mixed with PKH67 (2 ml) diluted in 500 ml Dilution C, and then incubated for 3 min at room temperature. An equivalent volume of 1% BSA was added to bind the excess PKH67. Exosomes labelled with PKH67 were collected as mentioned above, resuspended in neurobasal medium and added to neurons (cultured in 12-well plates). A Lactate Dehydrogenase (LDH) Assay Kit (Abcam, UK) was used to measure LDH levels in microglia to evaluate cellular damage after different treatments.

### RNA interference and transfection

P2X7R and CTSL knockdown experiments were performed by using primary microglia seeded in 10 cm dishes at 60%–80% confluence. The cells were transfected with P2X7R, CTSL or control small interfering RNA (siRNA) (scramble) (GenePharma, Shanghai, China) by using Lipofectamine™ RNAiMAX Transfection Reagent (Thermo Fisher, UK) according to the manufacturer’s instructions. The siRNA sequences were as follows: P2X7R siRNA: GCACAGUGAACGAGUAUUATT, UAAUACUCGUUCACUGUGCTT; CTSL siRNA: GGGCCUAUUUCUGUUGCUATT, UAGCAACAGAAAUAGGCCCTT; control siRNA (scramble): UUCUCCGAACGUGUCACGUTT, ACGUGACACGUUCGGAGAATT.

### Western blot analysis

Microglial medium was harvested, desalted, and concentrated by a Macrosep Advance centrifugal device with a molecular weight cutoff of 1 kDa (Pall Life Sciences, MI) to prepare cell supernatant samples. Microglial exosomes were isolated and purified by using an exosome isolation kit as described above. The protein concentration was determined by the Bradford protein assay. Proteins isolated from cell supernatants, whole-cell lysates and prepared exosomes were separated by SDS-PAGE (Millipore, Billerica, MA and Bio-Rad, Hercules, CA), the membranes were incubated overnight at 4 °C with primary antibodies. After washing, the membranes were incubated with horseradish peroxidase-linked anti-rabbit or anti-mouse secondary antibodies (1:20000, Jackson ImmunoResearch, USA) for 1.5 h at room temperature. The bands were visualised using chemiluminescence (BIO-RAD ChemiDoc MP Imaging System), and the density of each band was normalised to that of the loading control band, which was showed by silver staining using a ProteoSilver Silver Stain Kit (Sigma-Aldrich, USA). The loading control band was used to unify the loading quantity of exosome samples^65^. All blots were processed in parallel and derive from the same experiment.

### Coimmunoprecipitation (co-IP)

The interaction between extracellular α-Syn (oligomer and monomer) and P2X7R was assessed as previously described^4^. Briefly, precleaning was performed by the addition of 50 μl rProtein G agarose beads (Thermo Fisher, UK) and 2 μg anti-P2X7R, anti-human α-Syn, or species-relevant nonspecific mouse or rabbit immunoglobulin G (IgG). After 4 h of rotation at 4 °C, the beads were centrifuged at 1000 × *g* for 3 min and then washed with PBS 4 times. Primary microglia were harvested and lysed with co-IP lysis buffer (20 mM Tris-HCl (pH 7.4), 150 mM NaCl, 2 mM EDTA, 10% glycerol, and 0.5% Triton X-100) supplemented with 1:100 Halt protease and phosphatase inhibitor cocktail (Roche, Switzerland). The lysates were rotated at 4 °C for 30 min and cleared by centrifugation at 12,000 × *g*. Samples were then incubated with antibody-conjugated beads overnight at 4 °C. The proteins were separated by SDS-PAGE (10–12%) and electrotransferred onto polyvinylidene fluoride membranes. Western blot analysis was carried out as described above.

### CTSL activity assay

We harvested the supernatants of primary microglia and isolated exosomes in accordance with the above methods. CTSL activity was assessed by using an activity assay kit (Abcam, UK) according to the manufacturer’s instructions, and fluorometric intensity was measured in a white flat-bottom Costar 96-well plate (Corning, Lowell, MA) using a plate reader (Analyst^TM^ AD 96-384, Biosystems, USA) at an excitation wavelength of 400 nm and an emission wavelength of 505 nm. Background readings were subtracted from the sample values.

### Immunofluorescence analysis

To assess the localisation of CTSL and exosomes, and the colocalization between α-Syn oligomer and P2X7R, microglia were seeded on coverslips in 12-well plates, treated with 250 nM WT or A53T α-Syn oligomer for increasing amounts of time (0–6 h), washed three times in PBS, fixed with 4% paraformaldehyde and permeabilized with 0.1% Triton X-100. After blocking with 10% normal goat serum, the cells were incubated overnight at 4 °C with antibodies. After that, the cells were incubated with secondary antibodies conjugated to Alexa Fluor 488 or 546 (1:500, Molecular Probes, USA). Immunostaining was examined with a Nikon C2 + confocal microscope and photographed with a digital camera (CoolSNAP EZ, Photometrics).

### Neurotoxicity assays

Resuspended exosomes (extracted from microglia from each group) were added to primary neuronal cultures for 24 h. Protein extraction and subsequent western blotting were performed as described above. The membranes were incubated overnight at 4 °C with primary antibodies against caspase-3. For morphological analysis of neuronal damage, MAP2 immunostaining was examined under a Nikon Eclipse TE2000E fluorescence microscope. All obtained images were imported into Image-ProPlus software version 7.0 (Media Cybernetics, Silver Spring, MD), to quantify the levels of MAP2 staining. The assessors were blinded to the experimental groups during image acquisition and quantification.

### Statistical analysis

The data are expressed as the mean and standard deviation of at least three independent experiments. Analysis of the differences between group was performed by Student’s *t*-test. Significance was indicated by a *p*-value < 0.05. In all figures, the error bars represent the standard error of the mean. For all statistical analyses, measurements were taken from distinct samples.

## Supplementary information

Supplementary information file

Supplementary Figure Legend

## Data Availability

All data generated or analysed during this study are included in this published article (and its supplementary information files).
