# Supplementary material for: Cathepsin L-containing exosomes from α-synuclein-activated microglia induce neurotoxicity through the P2X7 receptor
Source: NPJ Parkinsons Dis. 2022 Oct 6;8:127. doi: 10.1038/s41531-022-00394-9 (PMC9537534; doi:10.1038/s41531-022-00394-9)

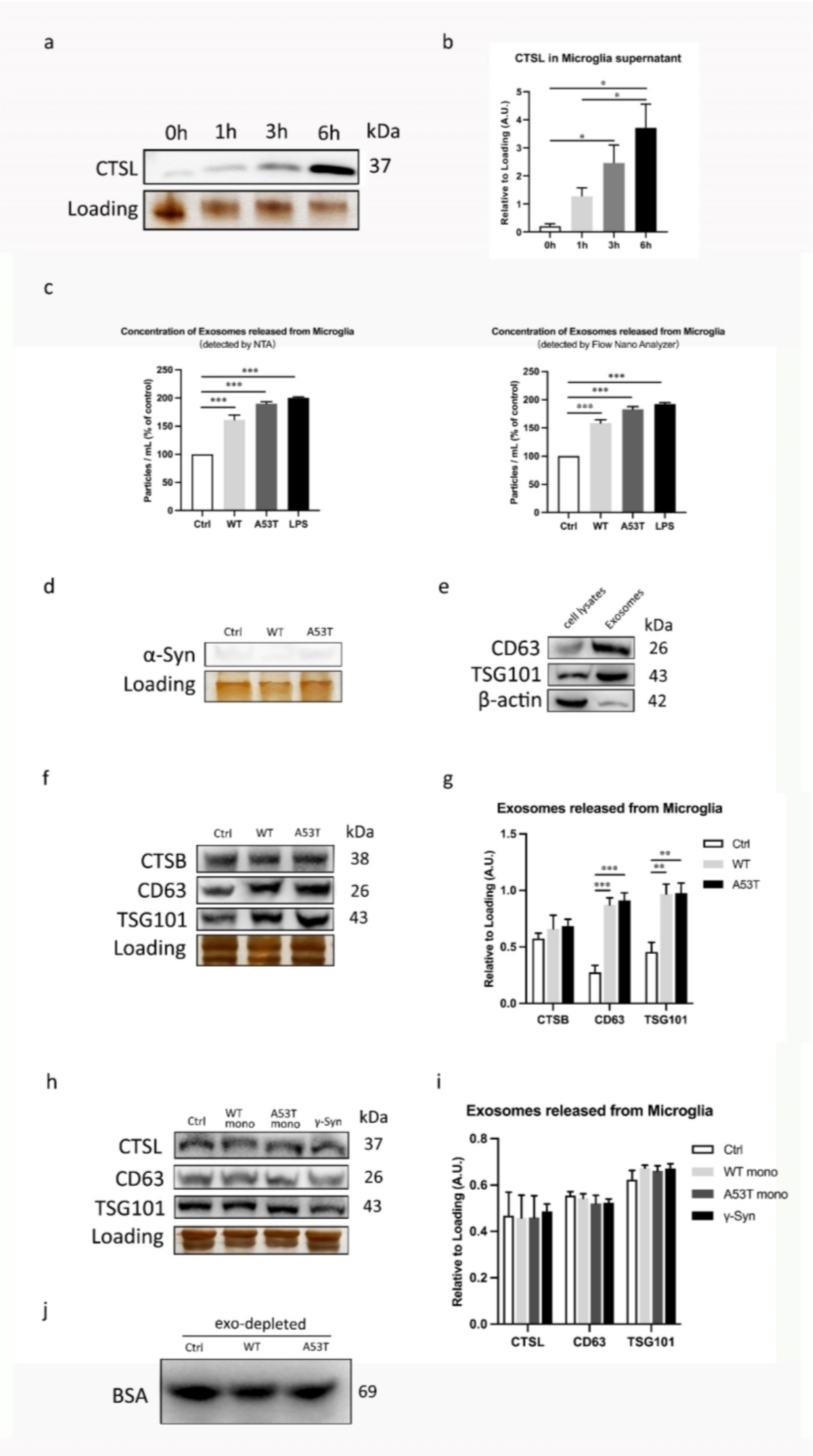

a
0h
1h
3h
6h
kDa
CTSL
37
Loading
b
CTSL in Microglia supernatant
Relative to Loading (A.U.)
0h
1h
3h
6h
c
Concentration of Exosomes released from Microglia
(detected by NTA)
Particles / mL (% of control)
Ctrl
WT
A53T
LPS
Concentration of Exosomes released from Microglia
(detected by Flow Nano Analyzer)
Particles / mL (% of control)
Ctrl
WT
A53T
LPS
d
Ctrl
WT
A53T
α-Syn
Loading
e
cell lysates
Exosomes
kDa
CD63
26
TSG101
43
β-actin
42
f
Ctrl
WT
A53T
kDa
CTSB
38
CD63
26
TSG101
43
Loading
g
Exosomes released from Microglia
Relative to Loading (A.U.)
Ctrl
WT
A53T
CTSB
CD63
TSG101
h
Ctrl
WT mono
A53T mono
γ-Syn
kDa
CTSL
37
CD63
26
TSG101
43
Loading
i
Exosomes released from Microglia
Relative to Loading (A.U.)
Ctrl
WT mono
A53T mono
γ-Syn
CTSL
CD63
TSG101
j
exo-depleted
Ctrl
WT
A53T
BSA
69

Supplementary Figure 1. a, b Western blot and quantitative analysis of CTSL release from the supernatant of microglia stimulated with LPS as a positive control for up to 6 hours (n=4); *$p < 0.05$. **c** Concentration of Exosomes released from Microglia detected by NTA and Flow Nano Analyzer. The quantitative data correspond to Fig. 1d, e; ***$p < 0.001$. **d, e** Determination of the purity of extracted exosomes. **d** Exosomes from microglia contained very little α-Syn protein. **e** Western blot analysis showing a small amount of exosomal markers CD63 and TSG101 present in cell lysates and the absence of the actin in exosomal lysates. **f, g** The results of Western blot analysis showing the level of CTSB in exosomes from WT and A53T α-Syn oligomer-activated microglia (n=3). **h, i** The results of Western blot analysis showing the level of CTSL in exosomes from γ-Syn, WT and A53T α-Syn monomers as a negative control (n=3). **j** BSA protein was detected in only exosomes-depleted supernatants of each group as loading control. Ctrl = control; Mono = monomer. Error bars represent s.e.m.

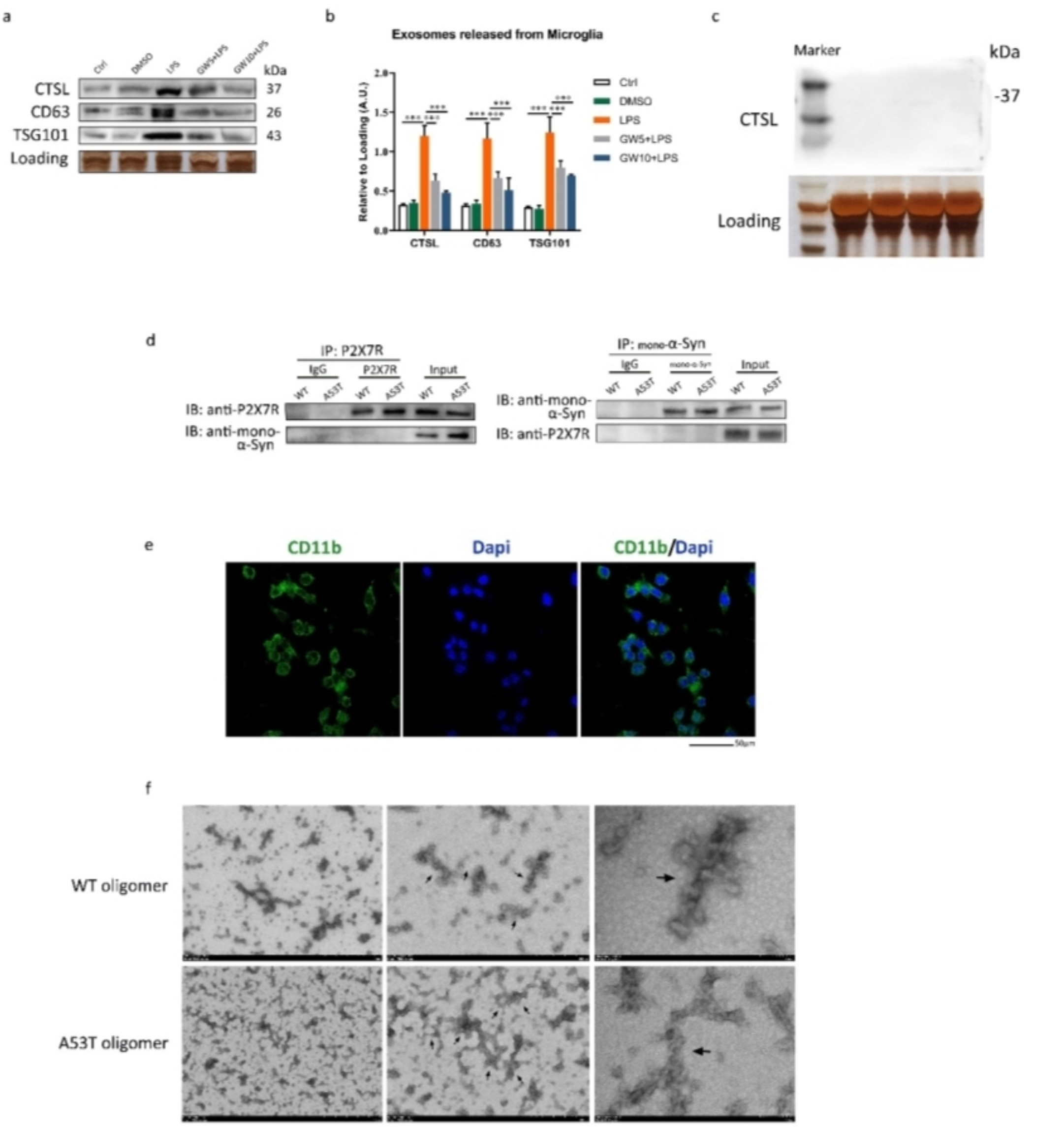

Supplementary Figure 2 a, b Western blot and quantitative analysis of CTSL release from microglia stimulated with LPS in the presence and absence of GW4869 (n=4); ***$p < 0.001$. Ctrl = untreated control; GW5 = 5 μM GW4869; GW10 = 10 μM GW4869. **c** BSA (250 nM) was employed as a negative control for microglial stimulation. Western blotting showed that CTSL was not released into the supernatant of BSA-treated microglia (n=3). **d** Western blot analysis of α-Syn expression in microglia after treatment with WT or A53T α-Syn monomer for up to 6 hours. Result showed there was no interaction between α-Syn monomer and

P2X7R. **e** Representative images of purified microglia detected by CD11b immunofluorescence staining. Scale bar: 50 μm. **f** The morphological observation by TEM of α-Syn aged for 7 days showing that the majority of α-Syn were protein aggregates (arrows). We provide images at different microscope multiples. Mono = monomer. Error bars represent s.e.m.

Original data of WB:

Figure 1

a

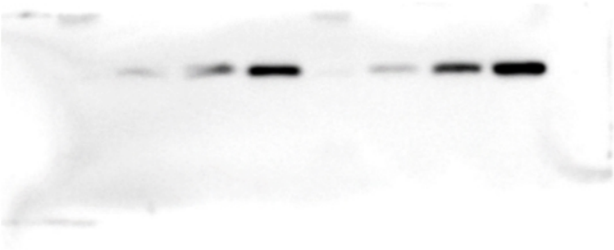

f

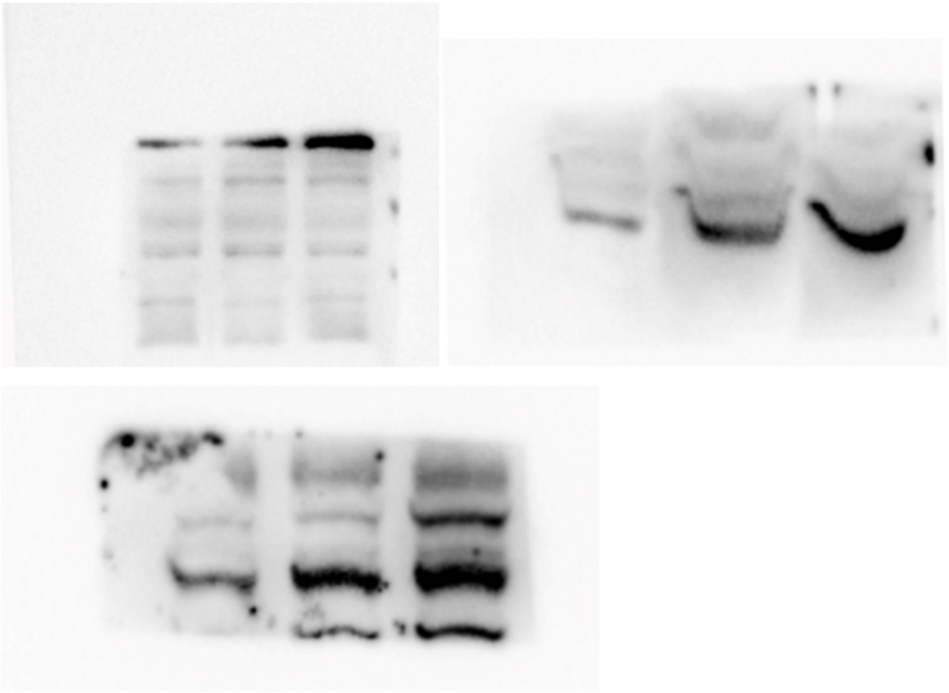

Figure2

b

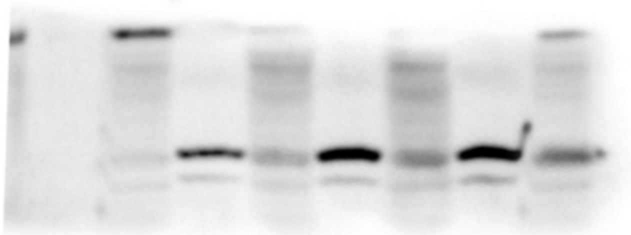

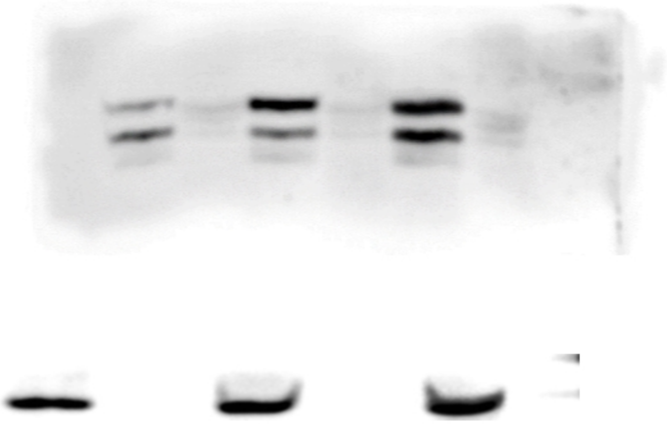

e

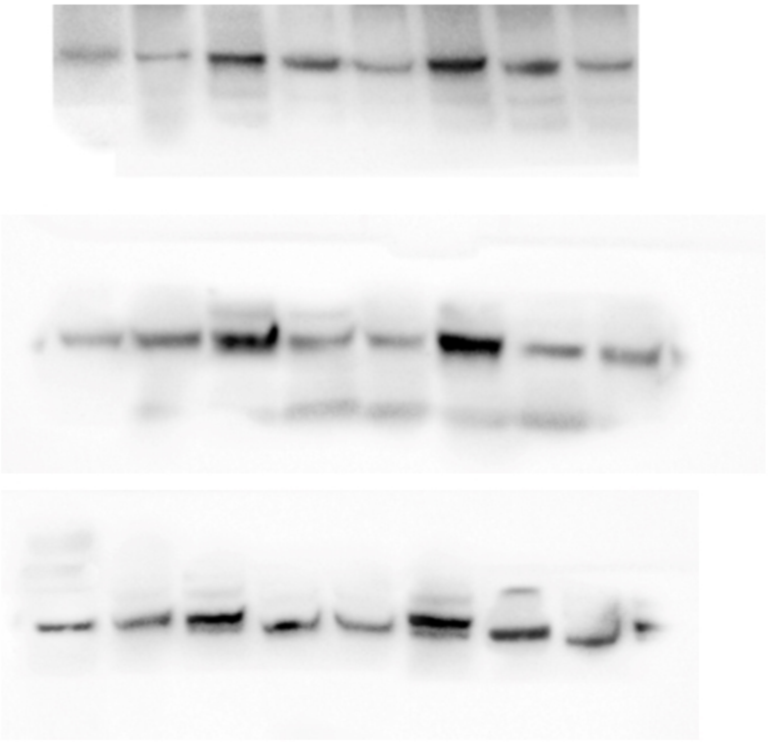

Figure3

a

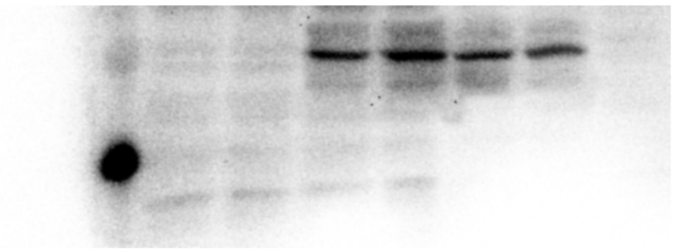

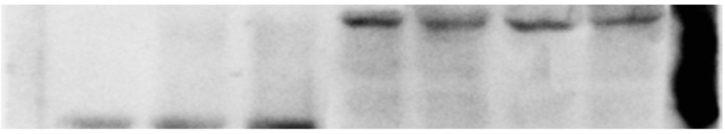

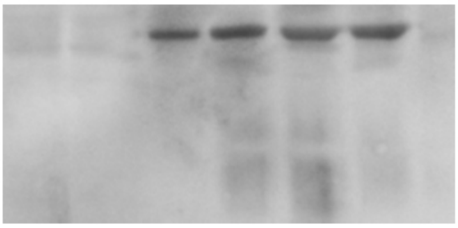

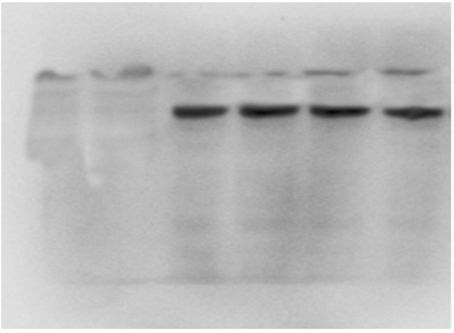

c

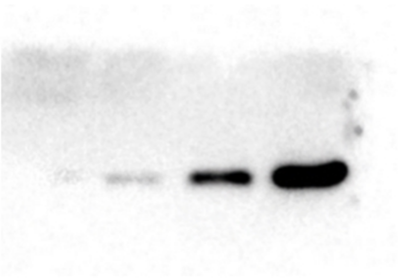

e

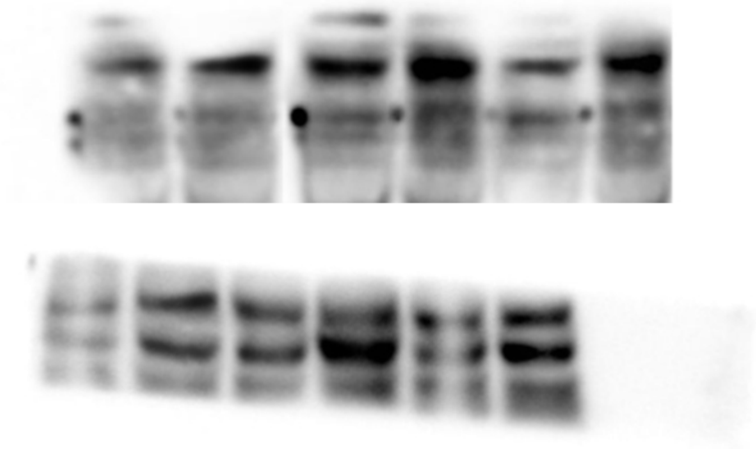

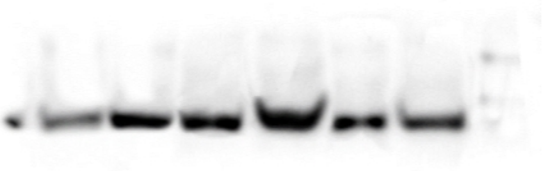

g

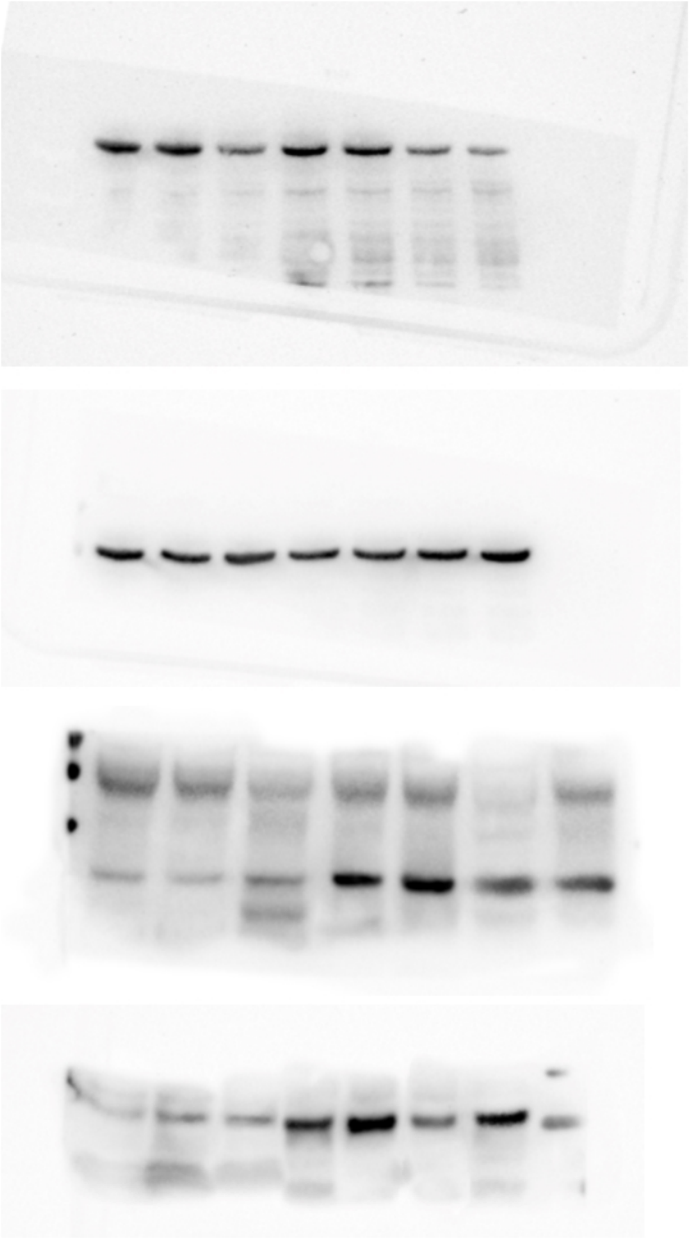

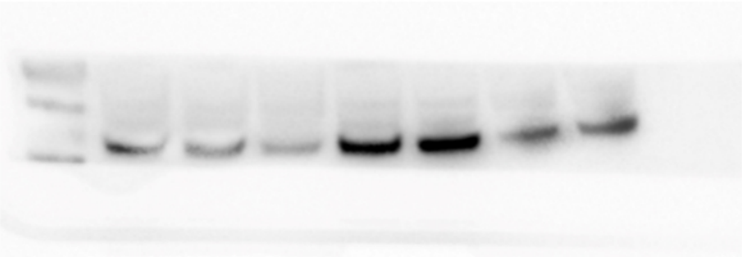

Figure4

a

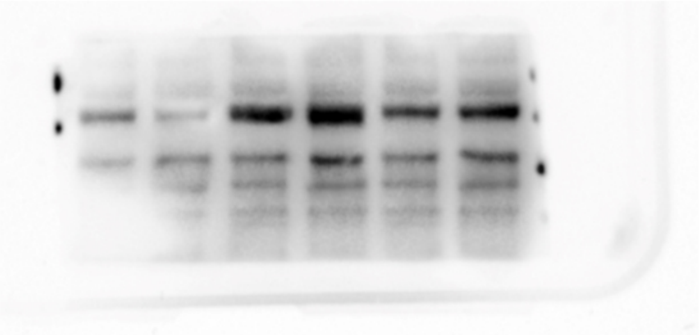

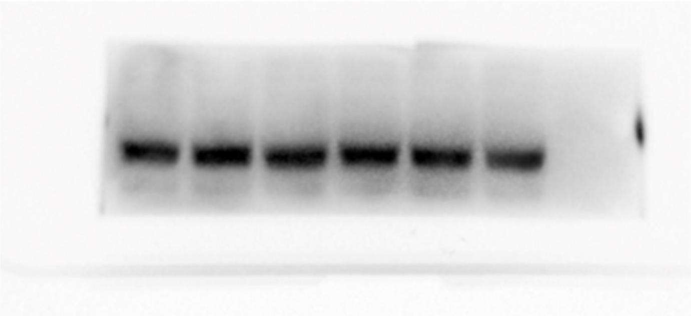

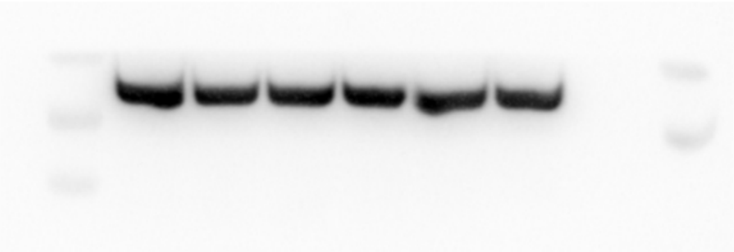

c

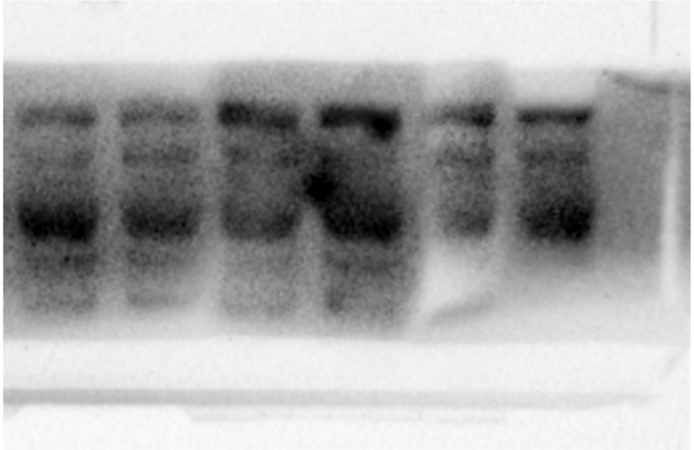

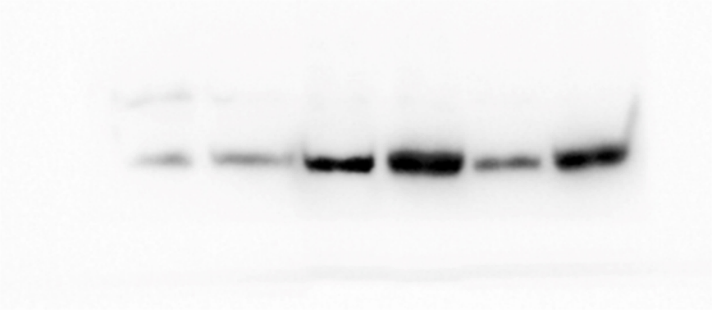

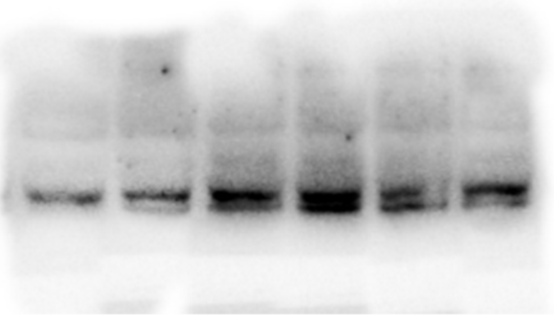

e

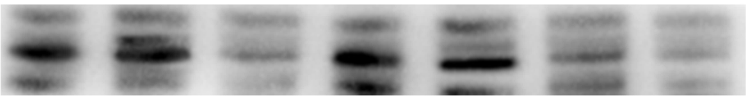

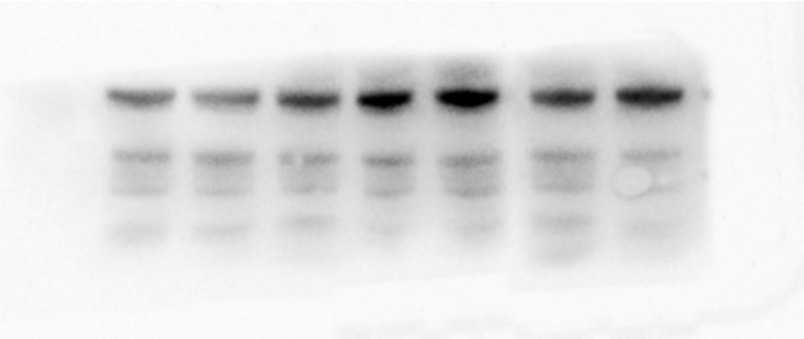

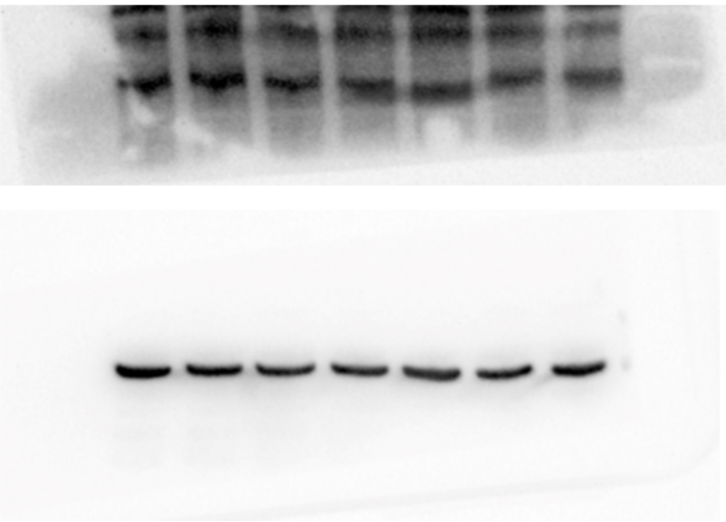

Figure5

a

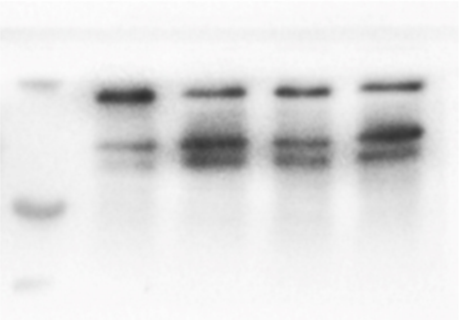

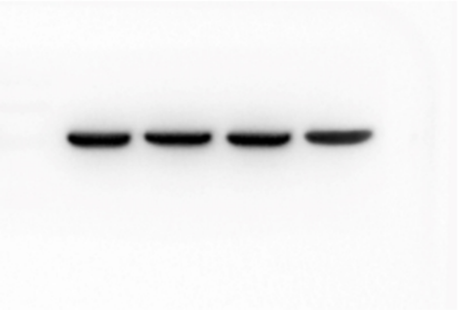

c

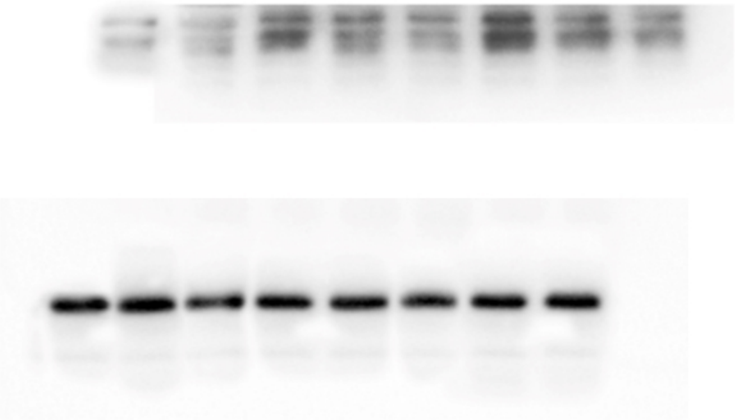

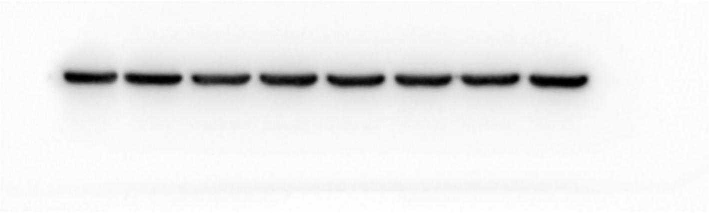

e

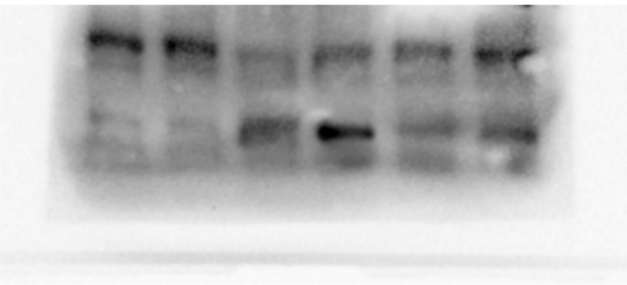

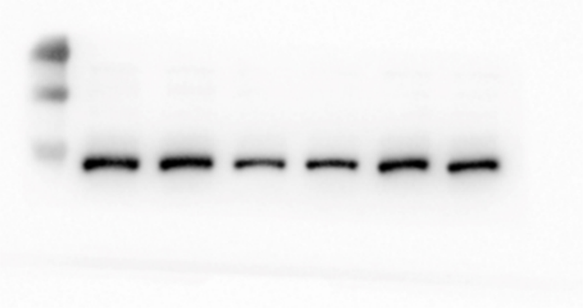

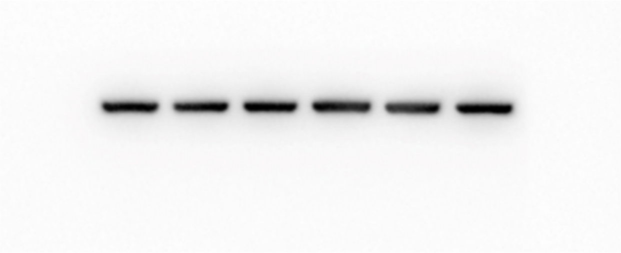

g

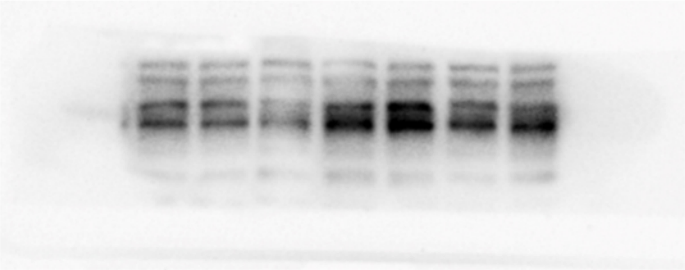

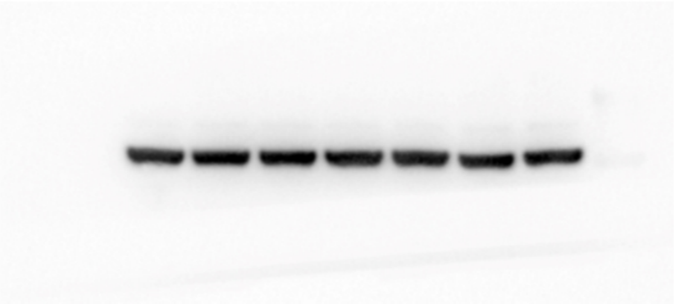

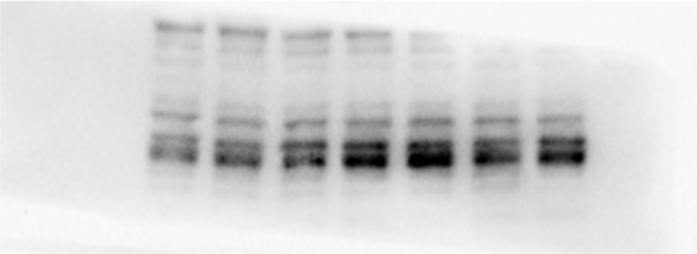

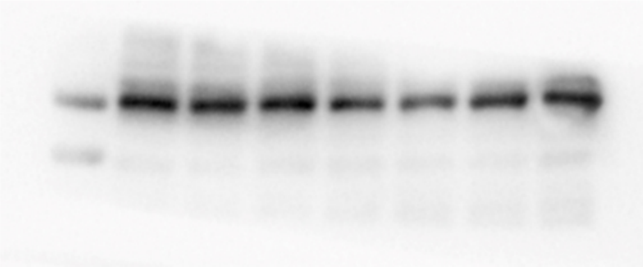

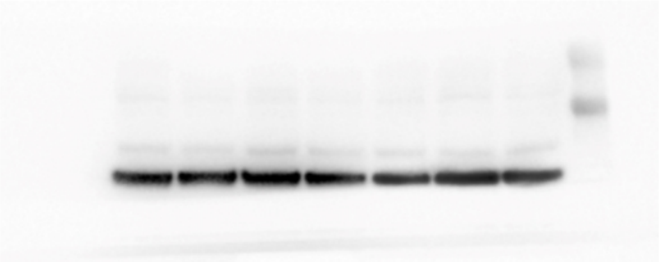

h

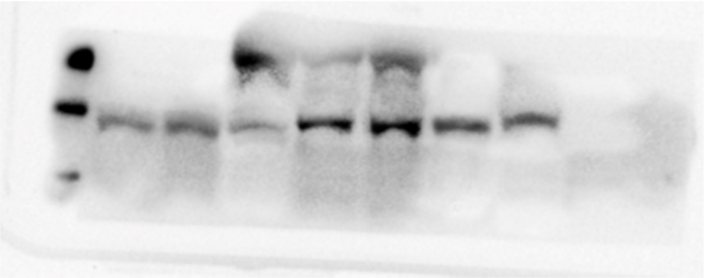

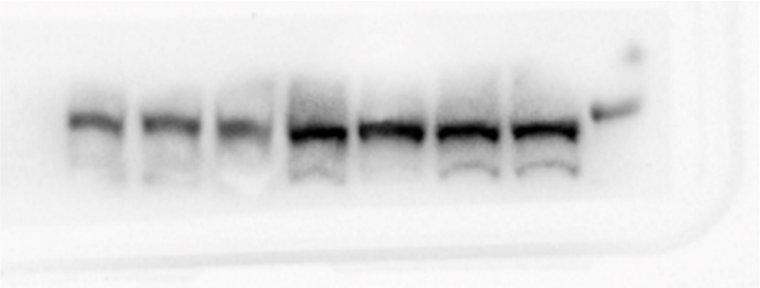

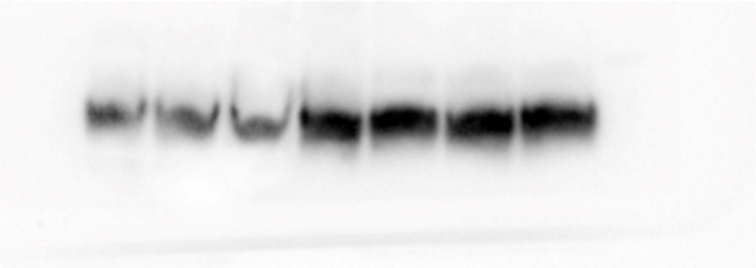

Figure7

b

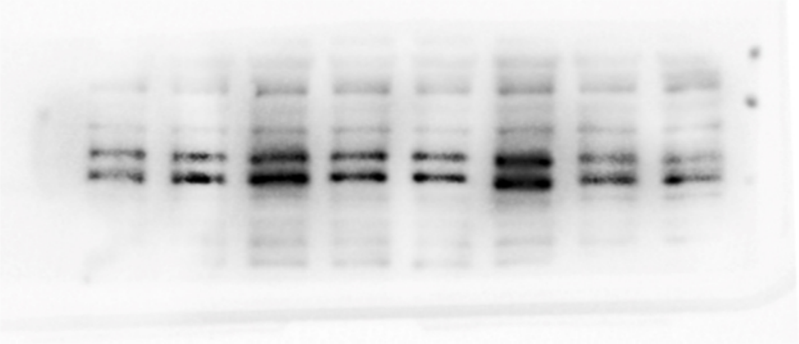

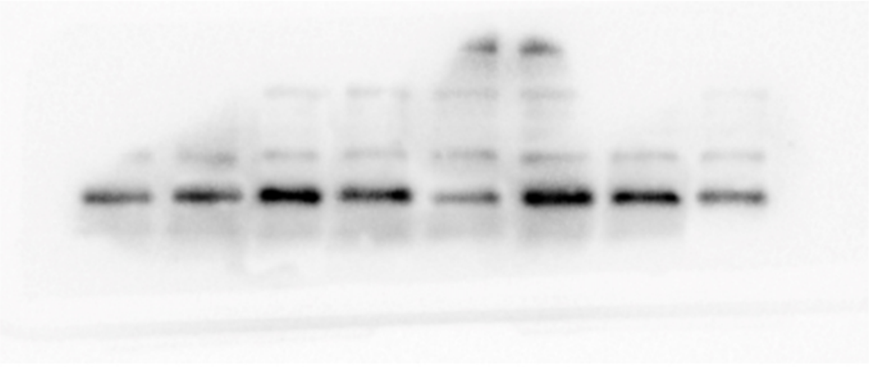

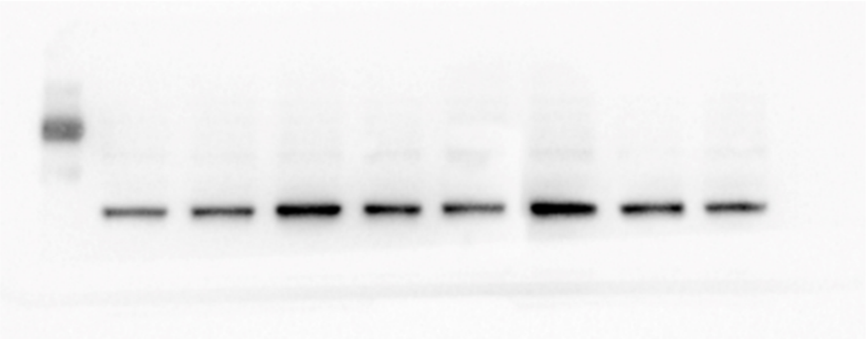

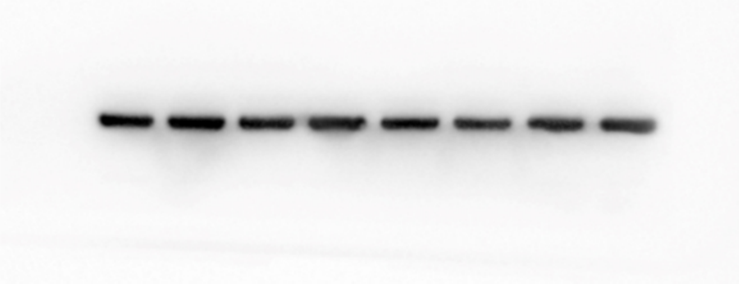

d

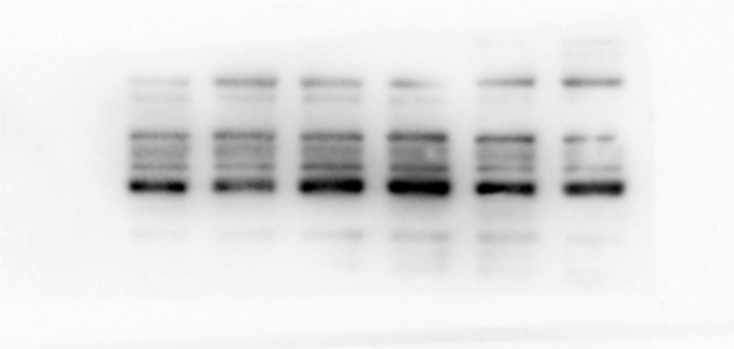

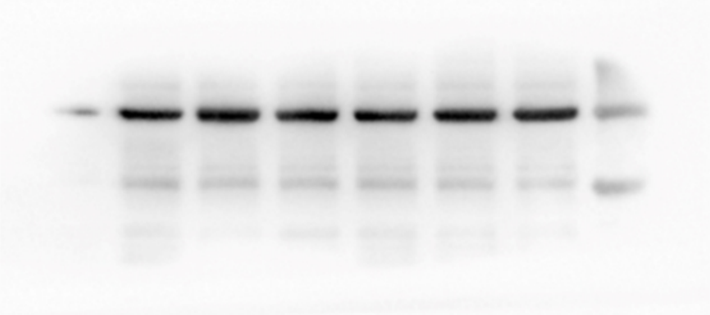

f

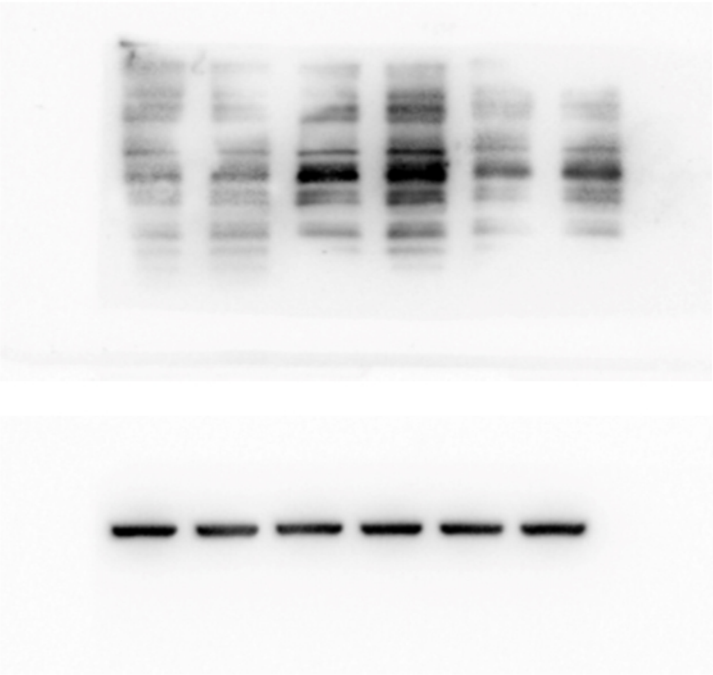

h

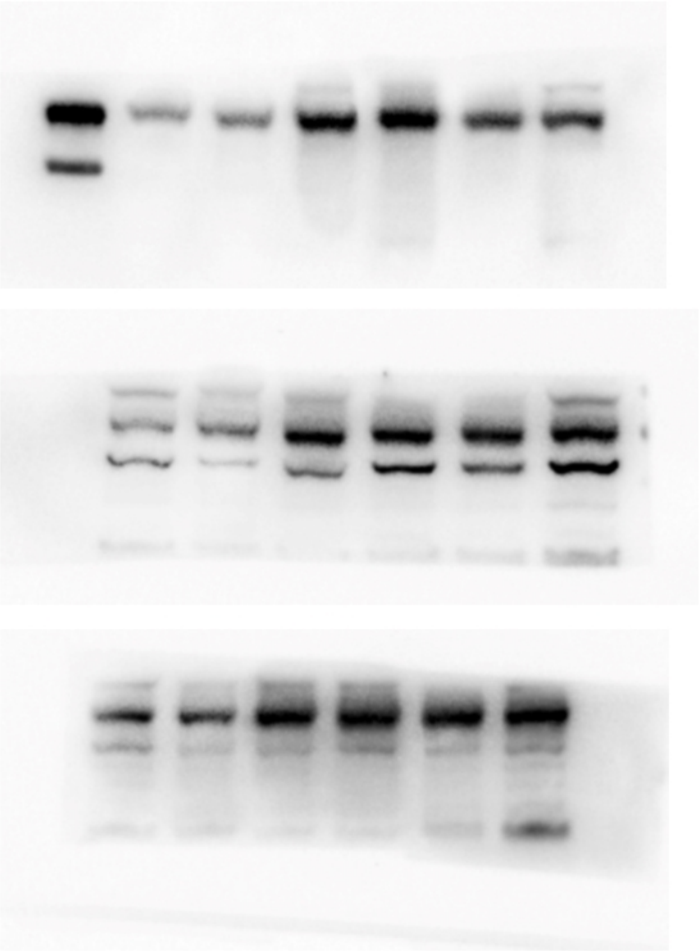

Supplementary Figure1

a

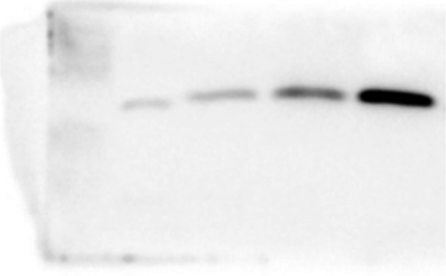

d

e

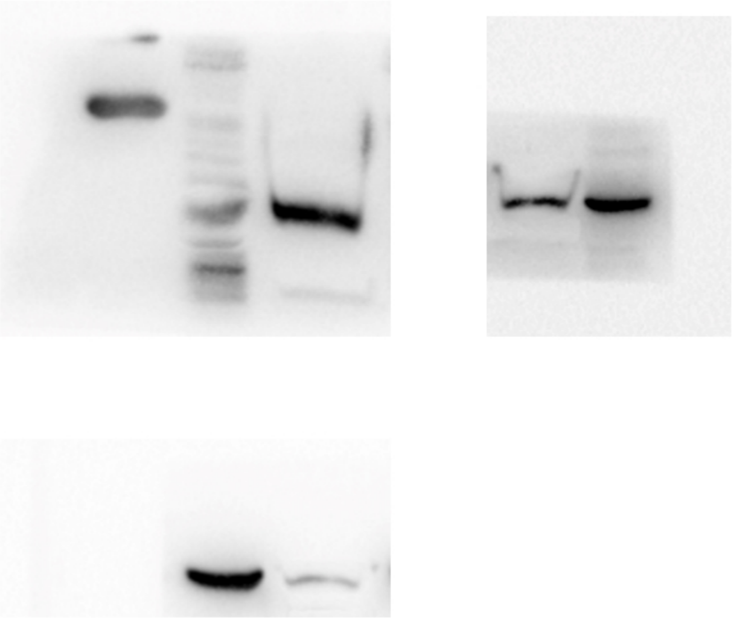

f

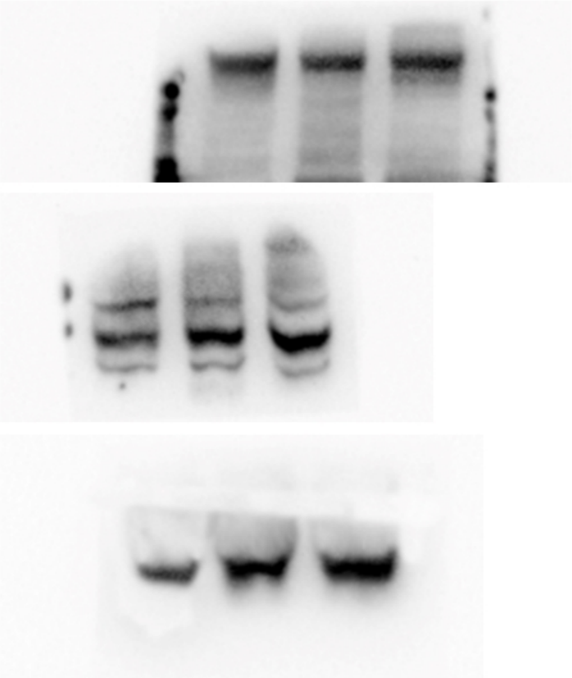

h

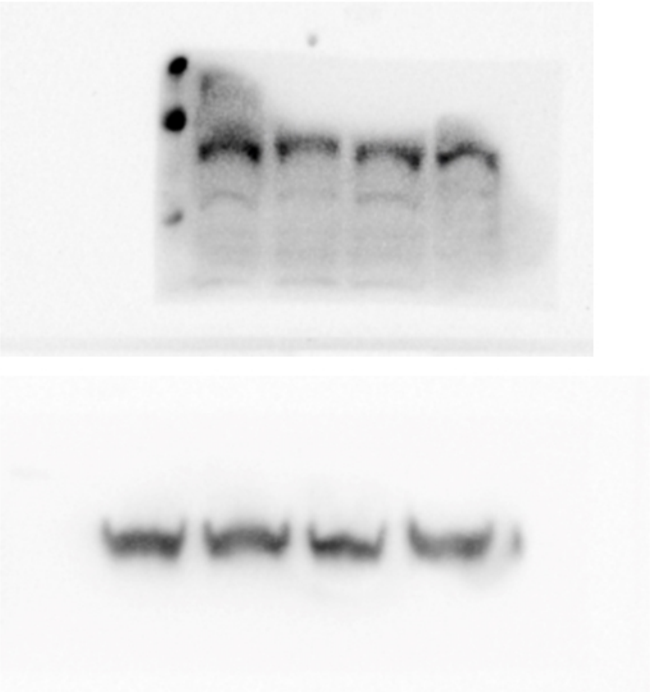

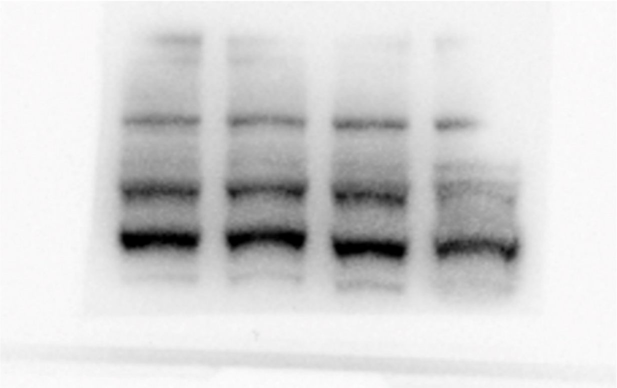

j

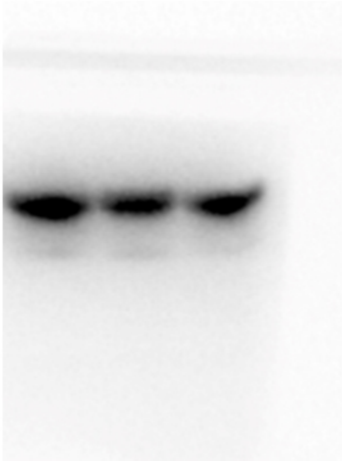

Supplementary Figure2

a

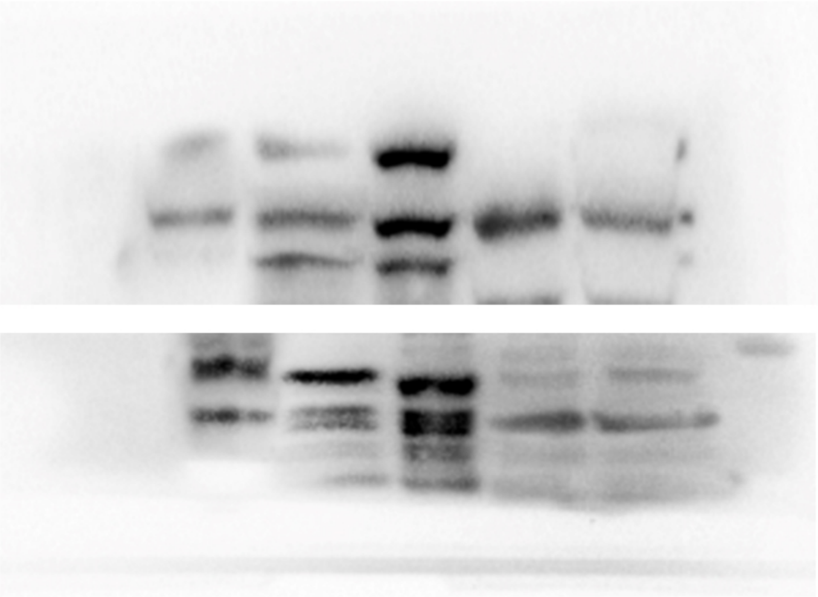

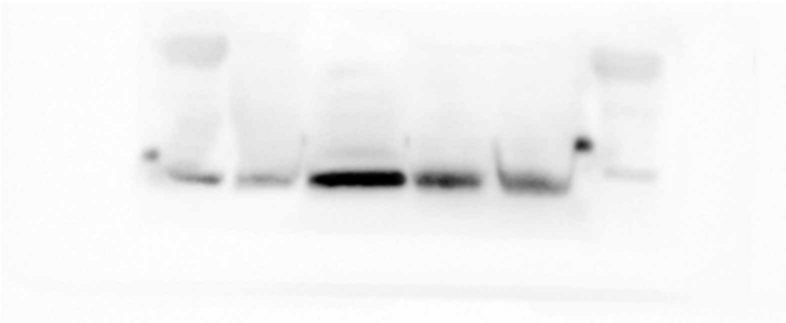

c

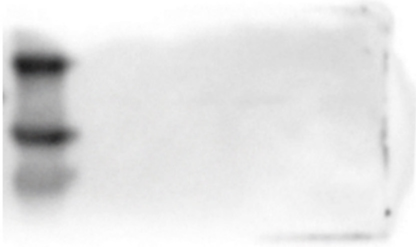

d

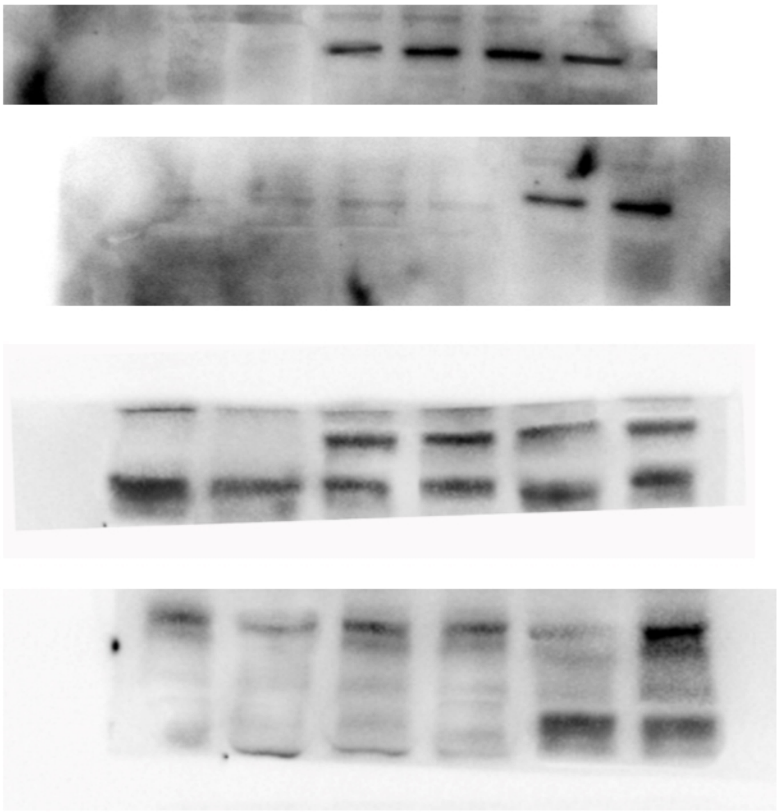

Supplement: Supplementary file 1 — Supplementary information file [file 41531_2022_394_MOESM1_ESM.pdf]
